## [Peer Review File · Nature Communications]

Reviewers' Comments:

Reviewer #1:

Remarks to the Author:

This manuscript by Choi et al reports the identification of a novel SLC17 isoform in the presynaptic nerve terminal of *C. elegans* neurons, which they named VST-1. They report that genetic ablation of VST-1 abolishes CO₂-avoidance of mutant animals, demonstrating that VST-1 is required for BAG-dependent chemotaxis. In such animals, glutamatergic signaling is enhanced, in agreement with antagonistic interactions between two SLC17 transporters. Loss of VST-1 results in less acidic synaptic vesicles. The authors conclude that VST-1 supports SV acidification by transporting an abundant anion substrate and that it competes for the electrochemical gradient required for glutamate uptake into synaptic vesicles.

I find this an interesting paper that addresses very important topics, i.e. the regulation of synaptic strength and the acidification of synaptic vesicles. While the proposed mechanisms are plausible, the conclusion is mainly based on indirect evidence. This is especially problematic as the mechanisms underlying SV acidification are not understood. I believe that it will mandatory to identify the anion substrate of VST-1 and the transport mechanisms of VST-1 to identify its cellular function.

Specific points.

1. I cannot find any sequence of VST-1, neither in the manuscript nor in any repository. I would also like to see an alignment of the VST-1 with the other members of the SLC17 family.
2. As the SLC17 family also encompass Na⁺ coupled phosphate transporters, I do not understand why the authors do not even discuss a potential cation transport by VST-1.
3. Mammalian VGLUTs are firmly established to also function as anion channels (Eriksen et al. (2016). *Neuron* 90:768-780). I would expect that such channel make an additional anion transport unnecessary for vesicle acidification. Could the authors comment on this.
4. Martineau et al. recently suggested that the VGLUT anion channel is necessary for exchange of synaptic vesicle Cl⁻ with glutamate to keep the SV membrane potential constant and to prevent osmotic gradients (Martineau et al. (2017). *Nat Comms* 8:2279). Such concepts should be discussed.
5. The authors report a SV pH value that significantly higher than in mammalian neurons. Is there agreement about this value in *C. elegans* in the literature?? Please discuss in more detail!
6. Please describe the experimental methods used for pH measurements in more detail.

Reviewer #2:

Remarks to the Author:

This is a very interesting study which uses a combination of genetics, behavior and imaging to demonstrate and characterize the role of a novel synaptic vesicle transporter. The work does not identify the substrate for this orphan transporter, but is generally convincing and provides considerable anatomic and physiological information to constrain its function. The work also appears carefully done, with almost all of the relevant controls and a dissection of the downstream circuitry. However, the scatter is surprisingly large for the avoidance index in particular—with much less variation for other behavioral variables. It would also be important to know that they can rescue the loss of VST-1. The paper is very well-written and the logic easy to follow.

Nonetheless, several surprising features leave questions about the mechanism. First, the increased glutamate release is consistent with uptake of another anion competing with EAT-4 for membrane potential, but the increased pH suggests that glutamate entry by EAT-4 has not entirely made up the difference—or pH would not have changed. It is also surprising that luminal pH does not change in the EAT-4 mutant—very similar to the mammalian VGLUTs—which suggests that VST-1 consumes more membrane potential than the canonical transporter, a point worth making in the text.

Second, VST-1 is required for CO₂ avoidance but does not have a dramatic effect on different aspects of the behavioral response to CO₂. The authors suggest that this may reflect a problem with integration of the response. However, the reduced baseline speed may simply make it difficult to observe CO₂ avoidance. It is also curious that this sensory system contributes to baseline

foraging. In addition, aspects of the behavioral response do not seem to be captured in the quantitation shown: the *eat-4* mutant shows slowing (more briefly than wild-type as the authors suggest) but the bigger effect seems to be rebound acceleration, which is not affected by *vst-1* mutation (Fig. 5b).

Third, VST-1 may affect more than glutamate. Clearly, many effects of the *vst-1* mutation involve glutamate: the increased turning response, which is consistent with increased glutamate release; occlusion of the *vst-1* avoidance phenotype by *eat-4* mutation; and rescue of *vst-1* with *glr-1* mutation. VST-1 localizes to neurons that are not apparently glutamatergic, where it might play a similar role in regulation of the driving force, but even in this system, the *vst-1* mutation reduces CO₂-evoked turning in the *eat-4* mutant, suggesting a role that may be independent of glutamate. In addition to suppressing the ectopic activation of glutamate receptors on RIA neurons, VST-1 may thus contribute to release of an independent signal.

In addition, it seems like AIB neurons are also affected by the *vst-1* mutation, at least from the representative traces.

Reviewer #3:

Remarks to the Author:

Choi et al. investigated mechanisms underlying glutamate signaling in *C. elegans*. They found that deletion of a vesicular transporter VST-1 increases glutamate release from the chemosensory BAG neuron, and that the excess release of glutamate recruits RIA interneurons inappropriately to the BAG-chemotaxis circuit. The authors further proposed that changes of the strength of glutamate synapses were due to altered levels of neurotransmitter packed into synaptic vesicles. Overall, this is an interesting study showing a functional link between altered synaptic transmission and its impact on circuit and behavior in living animals. However, there are several issues need to be addressed.

Main:

1) It is unclear how RIA is recruited in *vst-1* mutants. Does RIA express GLR-1? Brockie 2001 reported that RIA expresses GLR-2, GLR-3, and GLR-6, but not GLR-1. If this was the case, RIA recruitment by the BAG-dependent glutamate signaling would have to be indirect. This reviewer is confused how spillover of glutamate from BAG could alter RIA activity, if RIA does not have the essential GLR-1 receptor. This needs to be clarified. If the interaction between BAG and RIA are indirect, it would be important to identify the upstream neuron(s) that link BAG spillover to RIA activity, for example, through cell-specific rescue of *glr-1*, etc.

2) While the idea that VST-1 regulates glutamate loading into SVs is interesting, the evidence supporting this idea needs to be strengthened. For example, Figure 3h shows an increase in vesicular pH in *vst-1* mutants. It is unclear how much this pH change affects the interpretation of $\Delta F/F$, which was used to quantify vesicle release upon depolarization (Extended data Fig. 3d, e). In other words, it appears that the release of individual SVs in wt and *vst-1* mutants may not produce same unit values of $\Delta F/F$. If true, the same level of $\Delta F/F$ upon depolarization (Extended data Fig. 3d, e) may indeed be evidence for altered levels of SV fusion.

3) It is unclear whether *vst-1* mutations affect iGluSnFR expression and trafficking. Could the authors show a titration of glutamate using wild type and *vst-1* mutant neurons expressing iGluSnFR?

Minor:

4) Is there evidence showing that VST-1 and EAT-4 coexist on a synaptic vesicle? If this is too hard to show, the authors perhaps should discuss this point in the text.

5) Could the authors add discussion to explain why VST-1 does not affect AVA, AIB, and AIY

activities in response to BAG activation? Have the authors looked at GLR-1 trafficking and distributions in these neurons?

Response to Reviewers' Comments

Reviewers' comments are followed by our response in blue. We thank the reviewers collectively for their encouraging comments and focus here on responding point-by-point to their critiques.

Reviewer #1:

This manuscript by Choi et al reports the identification of a novel SLC17 isoform in the presynaptic nerve terminal of *C. elegans* neurons, which they named VST-1. They report that genetic ablation of VST-1 abolishes CO₂-avoidance of mutant animals, demonstrating that VST-1 is required for BAG-dependent chemotaxis. In such animals, glutamatergic signaling is enhanced, in agreement with antagonistic interactions between two SLC17 transporters. Loss of VST-1 results in less acidic synaptic vesicles. The authors conclude that VST-1 supports SV acidification by transporting an abundant anion substrate and that it competes for the electrochemical gradient required for glutamate uptake into synaptic vesicles.

I find this an interesting paper that addresses very important topics, i.e. the regulation of synaptic strength and the acidification of synaptic vesicles. While the proposed mechanisms are plausible, the conclusion is mainly based on indirect evidence. This is especially problematic as the mechanisms underlying SV acidification are not understood. I believe that it will mandatory to identify the anion substrate of VST-1 and the transport mechanisms of VST-1 to identify its cellular function.

The reviewer notes the significance of our study but raises the question of what substrate is transported by VST-1 and notes that detailed studies of VST-1 biophysics cannot be performed until this substrate is identified. Our study shows in multiple ways that VST-1 exerts its effects on neural circuits and behavior by controlling glutamatergic transmission, and this is our major conclusion. We agree that identifying substrates for VST-1 is an important and interesting goal given the role we have found for VST-1 in glutamatergic neurotransmission. We hope the reviewer agrees with us that our main conclusion - VST-1 regulates glutamatergic transmission *in vivo* - does not require the identification of a VST-1 substrate, and that an effort to identify such a substrate would constitute a new endeavor that is well beyond the scope of this study.

Specific points

1. I cannot find any sequence of VST-1, neither in the manuscript nor in any repository. I would also like to see an alignment of the VST-1 with the other members of the SLC17 family.

We apologize for not making more clear that VST-1 is a name we are proposing for a transporter currently named SLC-17.1. This is now stated more clearly in the manuscript (**lines 91-95**). We have also included the requested alignment of VST-1 to other members of the SLC17 family in a new figure, **Extended Data Fig. 1**.

2. As the SLC17 family also encompass Na⁺ coupled phosphate transporters, I do not understand why the authors do not even discuss a potential cation transport by VST-1.

We agree with the reviewer that the possibility that VST-1 can transport cations should be raised in the context of indicating alternative models for how VST-1 impacts glutamate transport. The model we propose focuses on anion transport, which is a hallmark of SLC17 transporters. We agree with the reviewer that the manuscript is improved by mentioning alternatives to our model, which we do in the Results and Discussion sections of the revised manuscript (**lines 212-214 and lines 350-352**).

3. Mammalian VGLUTs are firmly established to also function as anion channels (Eriksen et al. (2016). *Neuron* 90:768-780). I would expect that such channel make an additional anion transport unnecessary for vesicle acidification. Could the authors comment on this.

The reviewer raises an interesting point that highlights an important aspect of this part of our study. Our data indicate that in *C. elegans* sensory neurons loss of VGLUT does not cause a measurable change in vesicular pH (**Fig. 3h**). This is consistent with a model in which multiple vesicular transporters impact vesicular pH. Loss of VST-1, by contrast, does have a measurable effect on vesicular pH. These data are consistent with a model in which VST-1 mediates anion influx into vesicles and are not consistent with an alternative model in which VST-1 is a glutamate efflux transport. We have clarified our discussion of these data and the alternative models that we sought to distinguish by measuring vesicular pH in *C. elegans* sensory neurons (**lines 206-212**). We cannot speak directly to the question of whether the well described anion conductance associated with mammalian VGLUTs is a feature of *C. elegans* VGLUT, or to what extent such an anion conductance might impact vesicular pH. These are interesting questions, but we hope the reviewer agrees that they are not directly relevant to our main conclusions - that glutamatergic transmission within a neural circuit is regulated by functional interactions between VGLUT and another vesicular transporter.

4. Martineau et al. recently suggested that the VGLUT anion channel is necessary for exchange of synaptic vesicle Cl⁻ with glutamate to keep the SV membrane potential constant and to prevent osmotic gradients (Martineau et al. (2017). *Nat Comms* 8:2279). Such concepts should be discussed.

We thank the reviewer for bringing this interesting work to our attention. We agree that the manuscript is strengthened by incorporating more discussion of alternatives to the model that we propose and of the implications of interactions between vesicular transporters. We have expanded our discussion (**lines 350-354 and 357-361**) to more clearly state such alternatives and to put our study in the context of recent studies of VGLUT function and synaptic vesicle bioenergetics.

5. The authors report a SV pH value that significantly higher than in mammalian neurons. Is there agreement about this value in *C. elegans* in the literature?? Please discuss in more detail!

To our knowledge, there have been no prior measurements of vesicular pH in *C. elegans* neurons and the only comparison we can make is with data from elegant studies of mammalian neurons. We cannot say whether

differences in vesicular pH arise from species differences or from differences between types of glutamatergic neurons (we are measuring vesicular pH in chemosensory neurons while published data report pH of vesicles from interneurons and principal neurons of the CNS). We have revised the manuscript to make clear that our measurements indicate this difference in pH values and we briefly discuss potential explanations for this difference **(line 199-204)**.

6. Please describe the experimental methods used for pH measurements in more detail.

We apologize for the lack of clarity in our description of methods used to compute vesicular pH. The revised manuscript includes a more detailed description of the experimental methods and computations used to compute vesicular pH from measurements of synaptopHluorin fluorescence **(lines 593-602)**.

Reviewer #2:

This is a very interesting study which uses a combination of genetics, behavior and imaging to demonstrate and characterize the role of a novel synaptic vesicle transporter. The work does not identify the substrate for this orphan transporter, but is generally convincing and provides considerable anatomic and physiological information to constrain its function. The work also appears carefully done, with almost all of the relevant controls and a dissection of the downstream circuitry. However, the scatter is surprisingly large for the avoidance index in particular—with much less variation for other behavioral variables.

The variance of measured avoidance indices is consistent with prior experiments from our group and from others, and we hope that the reviewer agrees that variance notwithstanding the effects of mutations on chemotaxis behavior are clearly demonstrated by these data. The reviewer notes that parameters from other measured behaviors show less variance. This is likely because other behaviors are measured using high-throughput video tracking, which permits analysis of large numbers of animals and more accurate estimates of behavior parameters.

It would also be important to know that they can rescue the loss of VST-1. The paper is very well-written and the logic easy to follow.

We agree. We have measured chemotaxis of *vst-1* mutants carrying a transgene that encodes a VST-1::GFP fusion and we found that this transgene restores robust chemotaxis behavior to *vst-1* mutants. These data have been incorporated as **Figure 1f**.

Nonetheless, several surprising features leave questions about the mechanism. First, the increased glutamate release is consistent with uptake of another anion competing with EAT-4 for membrane potential, but the increased pH suggests that glutamate entry by EAT-4 has not entirely made up the difference—or pH would not have changed. It is also surprising that luminal pH does not change in the EAT-4 mutant—very similar to the mammalian VGLUTs—which suggests that VST-1 consumes more membrane potential than the canonical transporter, a point worth making in the text.

We agree. Data in **Figure 3h** indicate that synaptic vesicles in the chemosensory neurons that we studied harbor anion transporters other than EAT-4/VGLUT. Our observation that loss of VST-1 has a measurable impact on vesicular pH whereas loss of EAT-4/VGLUT does not is consistent with a model in which VST-1 supports more anion flux than EAT-4/VGLUT, as the reviewer states. We have consolidated and clarified our interpretation of these data per the reviewer's comment (**lines 322-361**).

Second, VST-1 is required for CO₂ avoidance but does not have a dramatic effect on different aspects of the

behavioral response to CO₂. The authors suggest that this may reflect a problem with integration of the response. However, the reduced baseline speed may simply make it difficult to observe CO₂ avoidance.

Video-tracking data shown in **Figure 5** indicate that *vst-1* mutants have defects in evoked turning, which is greatly increased compared to the wild type, and in their basal speed, which is less than that of the wild type. These effects are clear and significant. We apologize for any confusion caused by our description of these effects of VST-1 mutation. We have revised the section of the manuscript describing these results to make it more clear that loss of VST-1 causes these defects and that these defects are largely dependent on glutamate signaling (**lines 242-262**). With respect to the reviewer's second point, the reduced basal speed of *vst-1* mutants does not interfere with our ability to measure CO₂-avoidance. During chemotaxis assays, *vst-1* mutants disperse from the origin and at the end of the assay are distributed throughout the arena; they are not grossly defective in movement but instead fail to properly navigate away from the aversive CO₂ stimuli.

It is also curious that this sensory system contributes to baseline foraging. In addition, aspects of the behavioral response do not seem to be captured in the quantitation shown: the *eat-4* mutant shows slowing (more briefly than wild-type as the authors suggest) but the bigger effect seems to be rebound acceleration, which is not affected by *vst-1* mutation (Fig. 5b).

We agree that video tracking data shown in **Figure 5** contain many interesting features, and the reviewer notes several of these. Our purpose in measuring locomotory behavior at this resolution is to further assess interactions between *vst-1* and genes required for glutamate signaling. Specifically, we wished to determine whether VST-1 has functions other than to regulate glutamate signaling, in which case loss of VST-1 would have measurable effects on the behavior of *eat-4/VGLUT* mutants, and to confirm that effects of VST-1 mutation on behavior depend on the GLR-1 AMPA-type glutamate receptor. The data in **Figure 5** show that loss of EAT-4/VGLUT occludes many but not all effects of VST-1 mutation, and confirms that loss of GLR-1 restores behavior of *vst-1* mutants. These data bolster our argument that VST-1 is a regulator of glutamatergic transmission but also show that VST-1 likely has functions outside of glutamate signaling via AMPARs. We hope that our revised description of these data makes the hypotheses tested by these experiments and the basis for our conclusion more clear (**lines 242-262**).

Third, VST-1 may affect more than glutamate. Clearly, many effects of the *vst-1* mutation involve glutamate: the increased turning response, which is consistent with increased glutamate release; occlusion of the *vst-1* avoidance phenotype by *eat-4* mutation; and rescue of *vst-1* with *glr-1* mutation. VST-1 localizes to neurons that are not apparently glutamatergic, where it might play a similar role in regulation of the driving force, but even in this system, the *vst-1* mutation reduces CO₂-evoked turning in the *eat-4* mutant, suggesting a role that may be independent of glutamate. In addition to suppressing the ectopic activation of glutamate receptors on RIA neurons, VST-1 may thus contribute to release of an independent signal.

We agree with the reviewer's summary of our data and we thank the reviewer for this clear synopsis of our findings. Our data indicate that VST-1 is a regulator of glutamatergic signaling but might also function at synapses that use other transmitters. We apologize for not clearly communicating this conclusion in our initial manuscript, and we hope that our revised description of these data (**lines 242-262**) and our discussion of a potential role for VST-1 in non-glutamatergic signaling (**lines 427-434**) makes these points clearer.

In addition, it seems like AIB neurons are also affected by the *vst-1* mutation, at least from the representative traces.

The reviewer's comment prompted us to extend our study of AIB responses to BAG neuron activation. Our initial dataset did not reveal an effect of VST-1 mutation, but the sample size was relatively small. Even though we found strong evidence that ectopic activation of RIA neurons contributes to the phenotype of *vst-1* mutants, we agreed with the reviewer's suggestion to increase the number of measurements of BAG-to-AIB signaling. The revised manuscript now includes an expanded dataset of AIB responses (**Extended Data Fig. 7c**). This dataset confirms that in the wild type, BAG-to-AIB signaling is variable and, on average, AIBs do not acutely respond to BAG stimulation. The extended dataset reveals an effect of VST-1 mutation on BAG-to-AIB signaling: AIB neurons of *vst-1* mutants on average show a decrease in cell-calcium during BAG stimulation (**Extended Data Fig. 7c**). Because this effect of VST-1 mutation on AIB responses is unlikely to be mediated by the excitatory receptor glutamate GLR-1, which is required for the phenotype of *vst-1* mutants, we chose to not further characterize a role for AIB in the *vst-1* phenotype. The revised manuscript describes and discusses these new data (**lines 292-304 and lines 385-391**).

Reviewer #3:

Choi et al. investigated mechanisms underlying glutamate signaling in *C. elegans*. They found that deletion of a vesicular transporter VST-1 increases glutamate release from the chemosensory BAG neuron, and that the excess release of glutamate recruits RIA interneurons inappropriately to the BAG-chemotaxis circuit. The authors further proposed that changes of the strength of glutamate synapses were due to altered levels of neurotransmitter packed into synaptic vesicles. Overall, this is an interesting study showing a functional link between altered synaptic transmission and its impact on circuit and behavior in living animals. However, there are several issues need to be addressed.

Main:

1) It is unclear how RIA is recruited in *vst-1* mutants. Does RIA express GLR-1? Brockie 2001 reported that RIA expresses GLR-2, GLR-3, and GLR-6, but not GLR-1. If this was the case, RIA recruitment by the BAG-dependent glutamate signaling would have to be indirect. This reviewer is confused how spillover of glutamate from BAG could alter RIA activity, if RIA does not have the essential GLR-1 receptor. This needs to be clarified. If the interaction between BAG and RIA are indirect, it would be important to identify the upstream neuron(s) that link BAG spillover to RIA activity, for example, through cell-specific rescue of *glr-1*, etc.

We apologize for the confusion. RIA is known to express *glr-1*, and this is one reason why RIA was included in our study. Kuhara and colleagues report expression of *glr-1* in RIA interneurons (Kuhara, A. *et al.* (2008) *Science* 320, 803-807) and *glr-1* expression in RIA is clearly seen in single-cell RNAseq data collected by the CeNGEN consortium (Hammarlund *et al.* (2018) *Neuron* 99, 430-433). We have clarified this point in the text (lines 268-271).

2) While the idea that VST-1 regulates glutamate loading into SVs is interesting, the evidence supporting this idea needs to be strengthened. For example, Figure 3h shows an increase in vesicular pH in *vst-1* mutants. It is unclear how much this pH change affects the interpretation of $\Delta F/F$, which was used to quantify vesicle release upon depolarization (Extended data Fig. 3d, e). In other words, it appears that the release of individual SVs in wt and *vst-1* mutants may not produce same unit values of $\Delta F/F$. If true, the same level of $\Delta F/F$ upon depolarization (Extended data Fig. 3d, e) may indeed be evidence for altered levels of SV fusion.

The reviewer's point is well taken: changes in vesicular pH, if large enough, can alter the synaptophluorin signal. Because the shift in vesicular pH caused by loss of VST-1 is relatively small we did not believe that it would affect our interpretation of the synaptophluorin signal evoked by depolarization. The reviewer's comment prompted us to re-analyze our pHluorin data taking into consideration the slightly increased vesicular pH of *vst-1* mutant vesicles. As expected, the slight increase in vesicular pH in *vst-1* mutants did not alter our conclusion, and in fact brought the synaptophluorin signal from *vst-1* mutants into closer register with those from the wild type and *eat-4* mutants. We have added these adjusted traces to **Extended Data Figure 4** (formerly Extended Data Figure 3), where they are presented together with traces derived from the raw data.

We have added a detailed description of how the correction was performed to the Methods section (**lines 606-623**).

3) It is unclear whether *vst-1* mutations affect iGluSnFR expression and trafficking. Could the authors show a titration of glutamate using wild type and *vst-1* mutant neurons expressing iGluSnFR?

In principle, only drastic changes in expression of sensors such as iGluSNFR would skew the normalized fluorescence signal used to report glutamate release, and we never noticed any gross differences between the expression levels or localization of the iGluSNFR probe expressed in different mutants. However, the reviewer's question prompted us to quantify expression levels of iGluSNFR so we could confidently assert that they were comparable between genotypes. We have added the data as **Extended Data Figure 4c**, explained it in the Results (**lines 163-166**), and described how we quantified probe expression in the Methods section (**lines 551-555**).

Minor:

4) Is there evidence showing that VST-1 and EAT-4 coexist on a synaptic vesicle? If this is too hard to show, the authors perhaps should discuss this point in the text.

We agree that a direct demonstration of co-expression of EAT-4/VGLUT and VST-1 on synaptic vesicles would add to the manuscript. The reviewer's comment prompted us to analyze samples that co-express tagged VST-1 and tagged EAT-4 by immunogold-EM. We found examples of vesicular profiles that are double-positive for both transporters, suggesting that VST-1 and EAT-4 are co-expressed in synaptic vesicles. These data have been added to **Extended Data Figure 3** and are described in **lines 138-145** in the revised manuscript.

5) Could the authors add discussion to explain why VST-1 does not affect AVA, AIB, and AIY activities in response to BAG activation? Have the authors looked at GLR-1 trafficking and distributions in these neurons?

We have added some discussion of this interesting point to our revised manuscript (**lines 393-397**). The reviewer suggests one possible explanation for why loss of VST-1 affects some connections in the chemotaxis circuitry more than others. We hope the reviewer agrees that this interesting question would open a new line of investigation and go beyond the scope of this study.

Reviewers' Comments:

Reviewer #1:

Remarks to the Author:

The authors have satisfactorily addressed all my comments and I have no remaining concerns.

Reviewer #2:

Remarks to the Author:

The authors have addressed all of my concerns.

Reviewer #3:

Remarks to the Author:

The authors have fully addressed my concerns. Congratulations on a nice study.

REVIEWERS' COMMENTS

Reviewer #1 (Remarks to the Author):

The authors have satisfactorily addressed all my comments and I have no remaining concerns.

Reviewer #2 (Remarks to the Author):

The authors have addressed all of my concerns.

Reviewer #3 (Remarks to the Author):

The authors have fully addressed my concerns. Congratulations on a nice study.

We are happy that there are no more concerns to further address. We greatly appreciate the time and effort the editor and reviewers have put in to critically review our work.