# Supplementary material for: Opponent vesicular transporters regulate the strength of glutamatergic neurotransmission in a C. elegans sensory circuit
Source: Nat Commun. 2021 Nov 3;12:6334. doi: 10.1038/s41467-021-26575-3 (PMC8566550; doi:10.1038/s41467-021-26575-3)
Supplement: Supplementary file 1 — Supplementary Information [file 41467_2021_26575_MOESM1_ESM.pdf]

SUPPLEMENTARY INFORMATION

Opponent vesicular transporters regulate the strength of glutamatergic neurotransmission in a *C. elegans* sensory circuit

Jung-Hwan Choi, Lauren Bayer Horowitz, and Niels Ringstad

Supplementary Figure 1 page 1

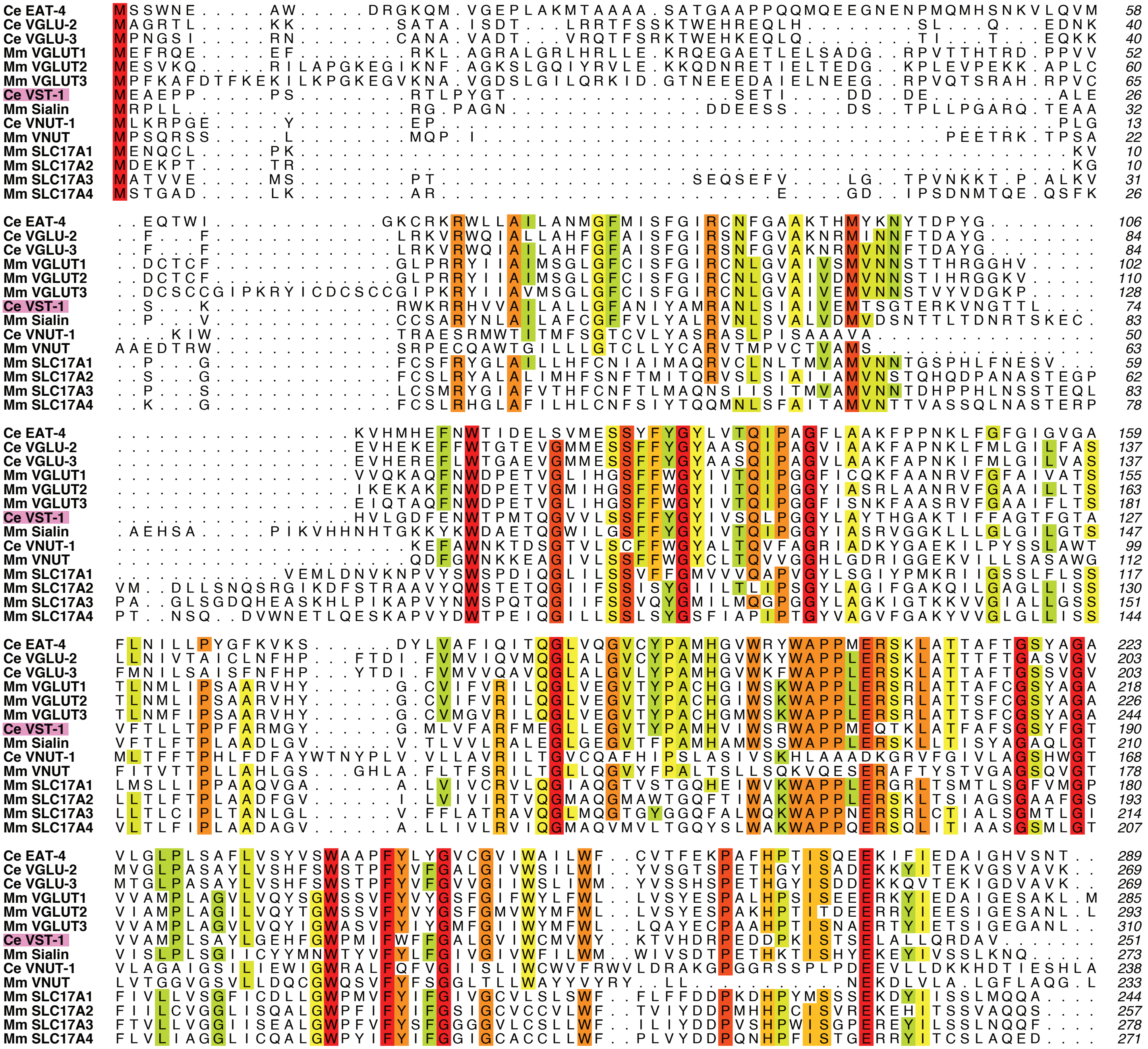

```
Ce EAT-4      .HPTIRSIPWKAIVTSKPVWAIIVANFARSWTFYLLLQNQLTYMKEALGMKIADSGLLAAIPHLVMGCVV  358
Ce VGLU-2     .NMTLTTLPWRDMMTSTAVWAIIICSFCRSWSFFLLLGNQLTYMKDVLHIDIKNSGLIAIFPQLGMCIVT  338
Ce VGLU-3     .NMSLTTLPWRDMMTSSAVWAIIICTFCRSWGFFLLLGNQLTYMKDVLHIDIKNSGFISIFPQFGMCIVT  338
Mm VGLUT1     NPVTKFNTPWRRFFTSMPVYAIIVANFCRSWTFYLLLISQPAYFEEVFGFEISKVGLVSALPHLVMTIIV  355
Mm VGLUT2     GAMEKFKTPWRKFFTSMPVYAIIVANFCRSWTFYLLLISQPAYFEEVFGFEISKVGMLSAVPHLVMTIIV  363
Mm VGLUT3     ASLSKFNTPWRRFFTSLPVYAIIVANFCRSWTFYLLLISQPAYFEEVFGFAISKVGLLSAVPHMVMTIVV  380
Ce VST-1      .SQNHYIVPWAQILRSKPVWAVIVAHSAQNLGFYIMLTNLPKMLKDIAGYNVEKAGIASSLPYFLMGFQI  320
Mm Sialin     .LSSQKVVPWGSILKSLPLWAIVVAHFSYNWSFYTLLTLLPTYMKEILRFNVQENGFLSALPYFGCWLCM  342
Ce VNUT-1     ATSPCPSVPWGTLFRHPAFWAAAVAQYTGGNSYSILFNWLPSYFHETFP..TAKGFVYNVVPSLAIVVTS  306
Mm VNUT       PVTKPSKVPWRQLFRKASVWAAICSQLCSACSFFILLSWLPTFFKETFP..NSKGWVFNVVPWMLAIPAS  301
Mm SLC17A1    .SSGRQSLPIKAMLKSLPLWAIILNSFAFIWSNSLLVTYTPTFISTVLHVNVRENGLLSSLPYLLAYICG  313
Mm SLC17A2    .SSPRRSVPIKAMVRCLPLWAIFMGFFSHFWLCTIIITYLPTYISTVLHVNIRDSGVLSSLPFIAASSCT  326
Mm SLC17A3    .SSEEQPLPIKAMLKSLPLWSMCLCTMTHQWLVNTFIMYTPTYISSVFKVNIRDNGFLSSLPFIVAWVLG  347
Mm SLC17A4    .CSLGWSLPIKAMVKSLPLWAIVVSYFCEYWLLSTVMAYTPTYISSVLQANLRDSGILSALPFMFGCVCI  340

Ce EAT-4      LMGGQLADYLRSNKILSTTAVRKIFNCGGFGGEAAFMLIVAYTT.SDTTAIMALIAAVGMSGFAISGFNV  427
Ce VGLU-2     LTSGQLSDYLRSSGKMSTEAVRKSVNTFGFTVEAVMLGCLAFVR.DPVIAVTFLIIACSGAGAVLSGFNV  407
Ce VGLU-3     LATGQLCDYLRSSGKMSTEAVRKSVNTFGFTVEAMMLGCLAFVR.DPVIAVTCLVIACTGSGSVLSGFNV  407
Mm VGLUT1     PIGGQIADFLRSRHIMSTTNVRKLMNCGGFGMEATLLLVVGYSH.SKGVAISFLVLAVGFSGFAISGFNV  424
Mm VGLUT2     PIGGQIADFLRSKQILSTTTVRKIMNCGGFGMEATLLLVVGYSH.TRGVAISFLVLAVGFSGFAISGFNV  432
Mm VGLUT3     PIGGQLADYLRSRKILTTTAVRKIMNCGGFGMEATLLLVVGFSH.TKGVAISFLVLAVGFSGFAISGFNV  449
Ce VST-1      ITGGQLCDYLRRDKHYDTLFVRKMACALGFIGQSVFLFLVMTTS.NSLLLVLFFSISIGLGGICWCGFSV  389
Mm Sialin     ILCGQAADYLRVKWNFSTISVRRIFSLVGMVGPAVFLVAAGFIGCDYSLAVAFLTISTTLGGFASSGFSI  412
Ce VNUT-1     LVAPVMASRALSEG.KTVTYTRKLMEGASLLGIAFCLMLVPMTS.SFWISLIIFTMAMAARGLHHGGVSV  374
Mm VNUT       LFSGFISDRLISQG.YRVITVRKFMQVMGLGLSSIFALCLGHTT.SFLKAMIFASASIGFQTFNHSGISV  369
Mm SLC17A1    ILAGQMSDFFLTRKIFSIVTVRKLFTTLGSFCPVIFIMCLLYLSYNFYSTVIFLTLANSTLSFSYCGQLI  383
Mm SLC17A2    ILGGQMADFLLSRNLLSLITVRKLFSSLGLLLPSLCAVALPFVTSSYIATIVLLILIPGTSNLCDSGFII  396
Mm SLC17A3    ILGGWLADFLLSKN.FRLITVRKFITLLGNAPPAALVAALPYIQSSYITTIIFLTISCGLCPLSQAGIYI  416
Mm SLC17A4    ILGGLLADFLLSRKILRLVTIRKLFTAVGVLASSGILLPLPWVRSSRSTTMAFLVLSSVFASLCDSGALI  410

Ce EAT-4      NHLDIAPRYAAILMGFSNGIGTLAGLTCPFVTEAFTAH.SKHGWTSVFLLASLIHFTGVTFYAVYASGEL  496
Ce VGLU-2     NHFDIAPRHAPILMGIANGLGAIAGVGG.IVTNSLTYQ.NPDGWQWVFLLAMSIDIFGIIFFLIFAKGDV  475
Ce VGLU-3     NHFDIAPRYAPILMGIANGLGAVAGVGG.MVTNTVTYQ.NPDGWKWVFLLAMAIDIFGVIFFLIFAKGDV  475
Mm VGLUT1     NHLDIAPRYASILMGISNGVGTLSGMVCPIIVGAMTKHKTREEWQYVFLIASLVHYGGVIFYGVFASGEK  494
Mm VGLUT2     NHLDIAPRYASILMGISDGVGTLSGMVCPIIVGAMTKNKSREEWQYVFLIAALVHYGGVIFYALFASGEK  502
Mm VGLUT3     NHLDIAPRYASILMGISNGVGTLSGMVCPLIVGAMTKHKTREEWQNVFLIAALVHYSGVIFYGVFASGEK  519
Ce VST-1      NHLDLAPQYAGHLMATSNTFATIPGIFGPLLVGAIVQNGTIGEWNVIMYIIISAYLLGAAIFWKFADATL  459
Mm Sialin     NHLDIAPSYAGILLGITNTFATIPGMTGPIIAKSLTPDNTIREWQTVFCIAAAINVFGAIFFTLFAKGEV  482
Ce VNUT-1     NPHDFAPNHAGSVFGVFNACGAITGFVGVYIAGHILEA.TNNNWSYVFVVTAAQCVVGAMVYTLLGTGQK  443
Mm VNUT       NIQDLAPSCAGFLFGVANTAGALAGVVGVCLSGYLIE..TTGSWTCVFHLVAIISNLGLGTFLVFGKAQR  437
Mm SLC17A1    NALDIAPRYYGFLKAVTALIGMFGGLISSTLAGLILNQDPEYAWHKIFFLMAGINVTCLVFYFLFAKGEI  453
Mm SLC17A2    NTLDVAPRYASFLMGISRGFGLTAGIISSTTTGFLISQVGHV........IEHLQVAGF...........  447
Mm SLC17A3    NALDIAPRYASFLMGTSRGLAHSSAVLVPIVAGFFLSQDSEFGWRNFFFVVFAVNLLGLIIYLVFGKADV  486
Mm SLC17A4    NFLDIAPRYAGFLKGLLQVFSYLAGGIAPTVAGFFISQDSEFGWRNVFFLAAAIDVVGLLFYLIFSRAEV  480

Ce EAT-4      QEWAEPKEEEEWSNKELVNKTGINGTGYGAAETTFT...QLPAGVDSSYQAQAAP....APGT...NP..  554
Ce VGLU-2     LPWAREPEKEETFN.EFVRRMSTMVR....SLSR.R...TRTRSTDTNYDKMEEERVNSSEMKACAK.SD  535
Ce VGLU-3     LPWAREPEKEETFN.EFVRRMSIKVR....SLSR.K...TRNREGDTSYEKMEED....SEMKPCSKKVE  532
Mm VGLUT1     QPWAEPEEMSEEKC.GFVGHDQLAGSDESEMEDE.AEPPGAPPAPPPSYGATHST...............  547
Mm VGLUT2     QPWADPEETSEEKC.GFIHEDELDE..ETGDITQ.N...YINYGTTKSYGATSQE....NGGW....P..  555
Mm VGLUT3     QDWADPENLSEDKC.GIIDQDELAE..ETELNHE.T...FVSPRKKMSYGATTQN....CEVQ....K..  572
Ce VST-1      QPWAAEHT.SFV......GQLE................................................  474
Mm Sialin     QSWALSDHHGHR.........N................................................  495
Ce VNUT-1     II....................................................................  445
Mm VNUT       VDLVPTHEDL............................................................  447
Mm SLC17A1    QDWAKEIKTTRL..........................................................  465
Mm SLC17A2    ......................................................................  447
Mm SLC17A3    QEWARERKLTRL..........................................................  498
Mm SLC17A4    QDWAKEPTFTHL..........................................................  492

Ce EAT-4      ........FASAWDEHGSSGVV....ENPH.YQQW.......  576
Ce VGLU-2     VPVPEEGVLNETQAIGGSLEVVP...EEPANKNLRTDRPEV.  573
Ce VGLU-3     ARAPAEKSESS.............................S.  544
Mm VGLUT1     .....................V....QPPRPPPPVRDY....  560
Mm VGLUT2     ..........NGWEK..KEEFVQEGAQDAYTYKDRDDY...S  582
Mm VGLUT3     ..........TEWRQ..QRESAFDGE.EPLSYQAEGDFSETS  601
Ce VST-1      ..........................................  474
Mm Sialin     ..........................................  495
Ce VNUT-1     ..........................................  445
Mm VNUT       ..........................................  447
Mm SLC17A1    ..........................................  465
Mm SLC17A2    ..........................................  447
Mm SLC17A3    ..........................................  498
Mm SLC17A4    ..........................................  492
```

Supplementary Fig. 1 Alignment of VST-1 with other SLC17 family members.

Sequence alignment of VST-1 with other SLC17 family members shown in Fig. 1d. Identical residues were colored from red to green by ALINE[1] indicating the fraction of aligned sequences which share the amino acid (red residues are shared by all of the aligned sequences).

Supplementary Figure 2

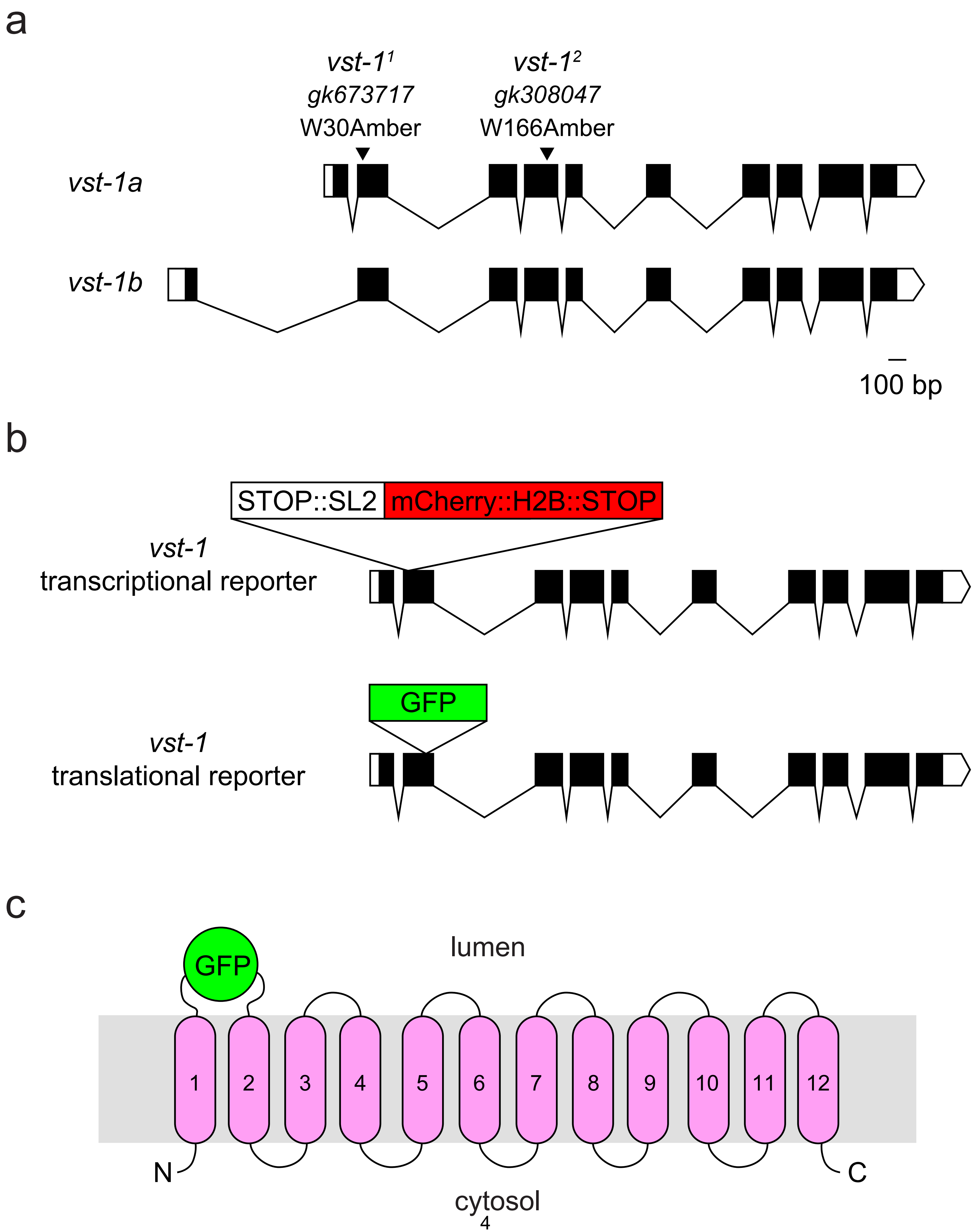

Supplementary Fig. 2 Alleles of *vst-1* and reporters of VST-1.

a Alleles of *vst-1* used in this study. Arrowheads indicate locations of nonsense mutations of each allele in the second and fourth exons, respectively, of both isoforms of *vst-1*. **b** Schematic of the *vst-1* transcriptional fosmid reporter, *vst-1 fosmid::stop::SL2::1xNLS::mCherry::H2B::stop* (pEH62) and the *vst-1* translational reporter, *vst-1::gfp* (pJC75 and pJC106). **c** Schematic of the VST-1::GFP fusion protein indicating the GFP insertion site within the first intraluminal loop.

Supplementary Figure 3

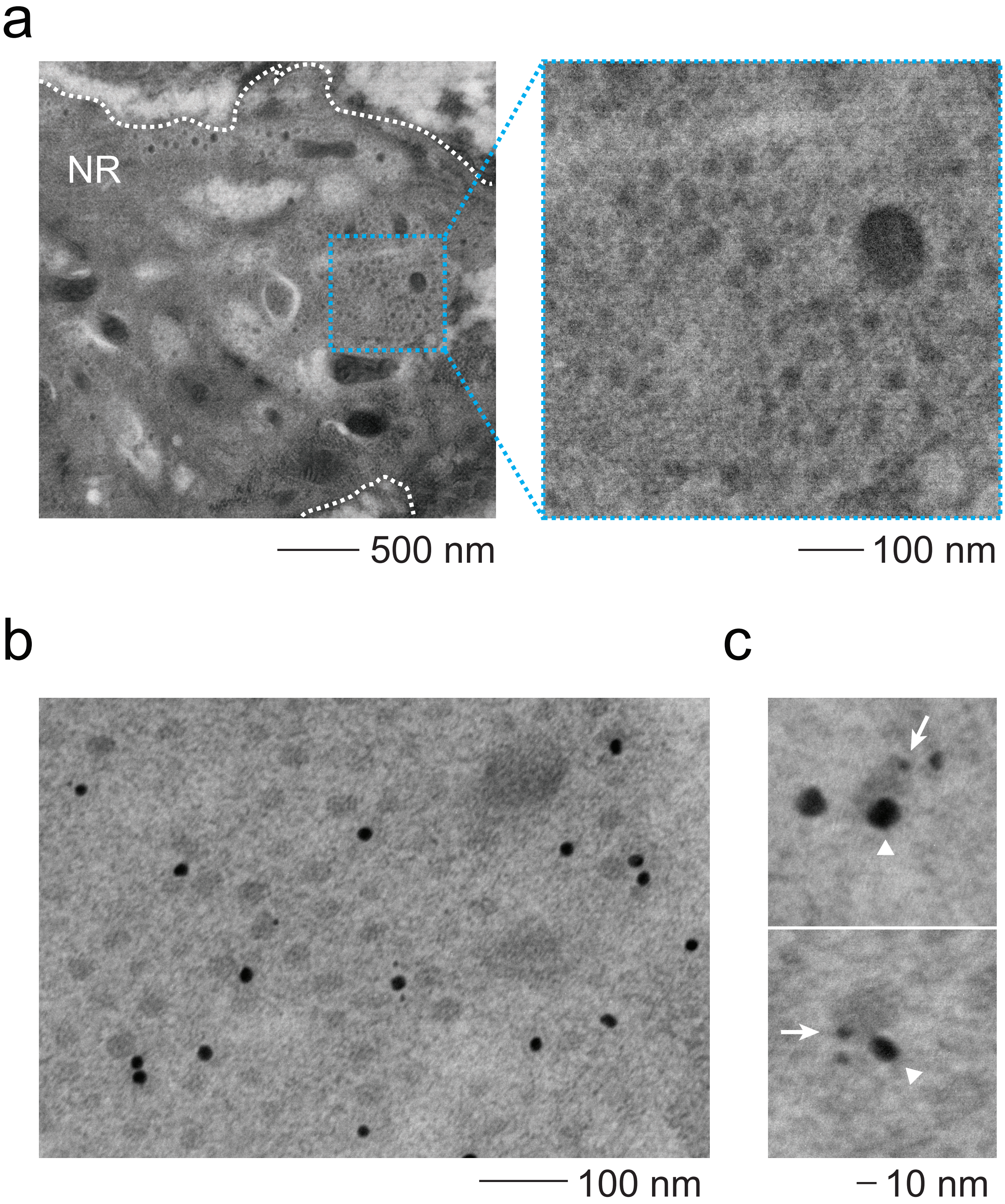

Supplementary Fig. 3 Controls for immunogold electron microscopy and double-immunolabeling of the nerve ring of a strain expressing VST-1::GFP and EAT-4::mCherry.

a Electron micrograph of a section stained with PA15 secondary antibody only (no primary anti-GFP antibody). The left panel shows a larger field of view at a magnification of 31000x. The boundary of the nerve ring (NR) is marked by a dashed white line. The right panel shows a magnified view of the region indicated by dashed cyan lines. The micrograph is representative of images from two different regions of the nerve ring. **b**-**c** show section stained with anti-GFP/ PA15 (Protein A 15nm gold-conjugated) antibodies and anti-RFP/ PA5 (Protein A 5nm gold-conjugated) antibodies. 15nm gold particles indicate the location of VST-1::GFP and 5nm gold particles indicate that of EAT-4::mCherry. The micrographs are representative of six synaptic vesicles (from five different regions of the nerve ring) associated with both VST-1 and EAT-4. **b** Electron micrograph showing distribution of large and small immunogold particles indicating VST-1 and EAT-4, respectively, at a magnification of 53000x. **c** Electron micrographs showing a synaptic vesicle associated with immunogold particles indicating VST-1 (arrowhead) and EAT-4 (arrow) at a magnification of 88000x.

Supplementary Figure 4

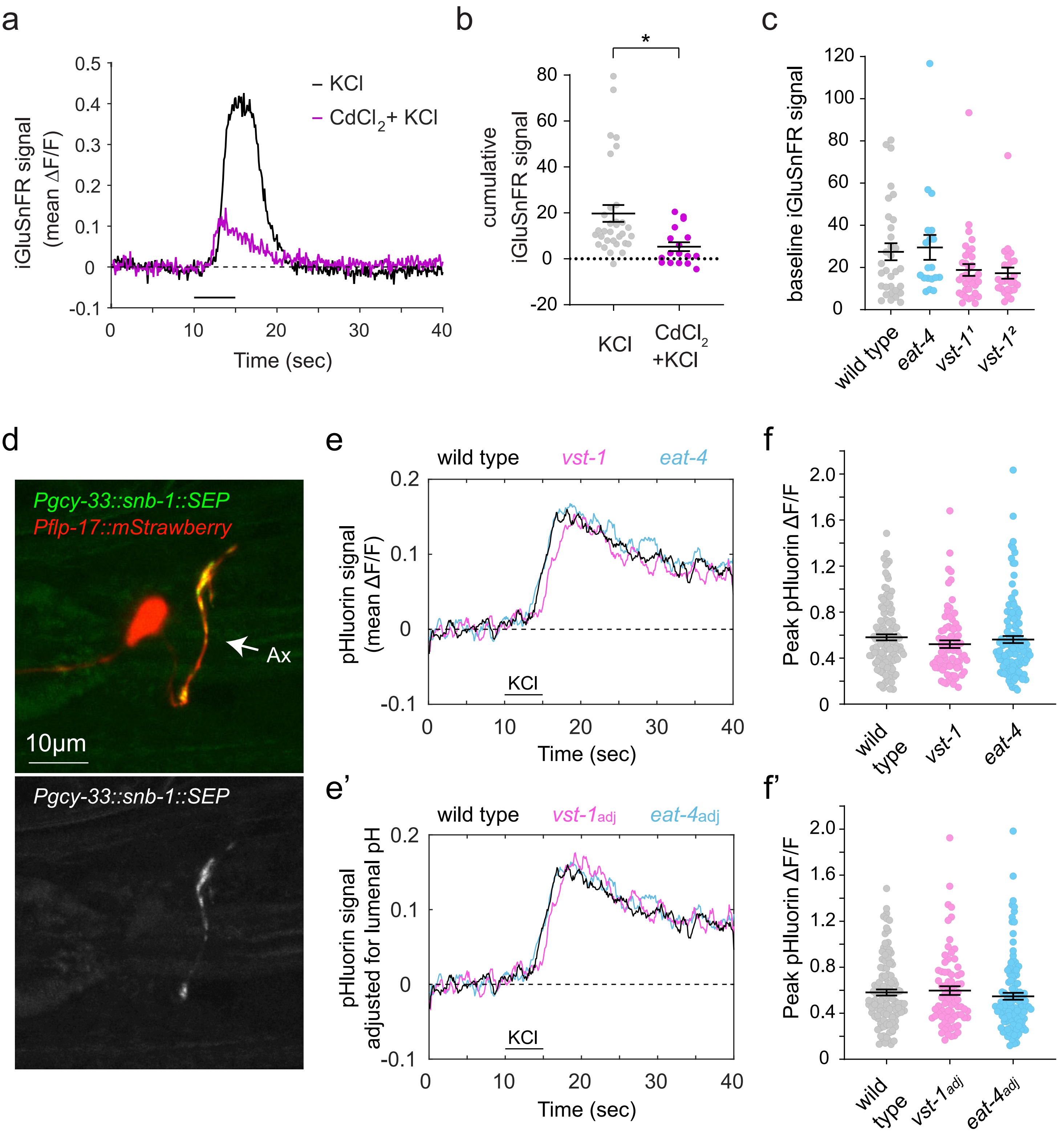

Supplementary Fig. 4 Characterization of iGluSnFR and synaptopHluorin in BAG neurons.

a Mean iGluSnFR signal (mean ΔF/F) of cultured wild type BAG neurons stimulated by KCl in the presence and absence of $CdCl_2$. The mean trace for KCl only was replotted from Fig. 3a. **b** Cumulative iGluSnFR signal (area-under-the-curve of ΔF/F between 10-20 sec, mean ± SEM) in the presence (n = 17) and absence (n = 32) of $CdCl_2$. Asterisk indicates a statistical difference (P = 0.0012, two-sided, Mann-Whitney test). (**c**) Level of baseline iGluSnFR expression (mean ± SEM) of cells analyzed in Fig. 3f (n = 32, 19, 34, and 26 for wild type, *eat-4*, *vst-1*^{1}, and *vst-1*^{2}, respectively). For each neuron, the mean fluorescence prior to KCl application (0-10 sec) (in arbitrary fluorescence units) was divided by the excitation light intensity (% maximum intensity) to adjust for the difference in excitation light intensity used. There were no statistically significant differences among genotypes (Kruskal-Wallis test, Dunn's test for multiple comparisons between wild type and other genotypes, P values for each comparison - wild type vs *eat-4*: > 0.9999; wild type vs *vst-1*^{1}: 0.47; wild type vs *vst-1*^{2}: 0.37). **d** Micrograph showing the synaptopHluorin strain (*Pgcy-33::snb-1::superecliptic pHluorin; Pflp-17::mStrawberry; Punc-122::mCherry*) dissociated to obtain cultured BAG neurons. SynaptopHluorin expressed specifically in BAG neurons is enriched in puncta along the BAG axon indicating synaptic localization. **e** The plot shows mean synaptopHluorin signal (mean ΔF/F) of KCl-responsive puncta in response to 100 mM KCl after passing individual traces through a 1 sec moving-average filter. **e'** The plot shows the mean synaptopHluorin signal adjusted for luminal pH. Individual traces were adjusted by multiplying an adjustment factor for the genotype (described in Methods) and the mean trace for each genotype is shown in this plot. **f** Maximum synaptopHluorin signal (maximum ΔF/F between 10-20 sec, mean ± SEM) from KCl-responsive puncta from cultured wild type, *vst-1(gk308047),* and *eat-4(ky5)* BAG neurons (n = 118, 76, and 115, respectively). Quantification was conducted on the raw traces. There were no statistically significant differences among the genotypes (Kruskal-Wallis test, Dunn's test for multiple comparisons between wild type and other genotypes, P = 0.15 for wild type vs *vst-1* and P = 0.48 for wild type vs *eat-4*). **f'** Quantification of the maximum signal (mean ± SEM) when adjusted for luminal pH. There were no statistically significant differences among the genotypes (Kruskal-Wallis test, Dunn's test for multiple comparisons between wild type and other genotypes, P > 0.9999 for wild type vs *vst-1* and P = 0.24 for wild type vs *eat-4*, n = 118, 76, and 115 for wild type, *vst-1*, and *eat-4*, respectively).

Extended Data Figure 5

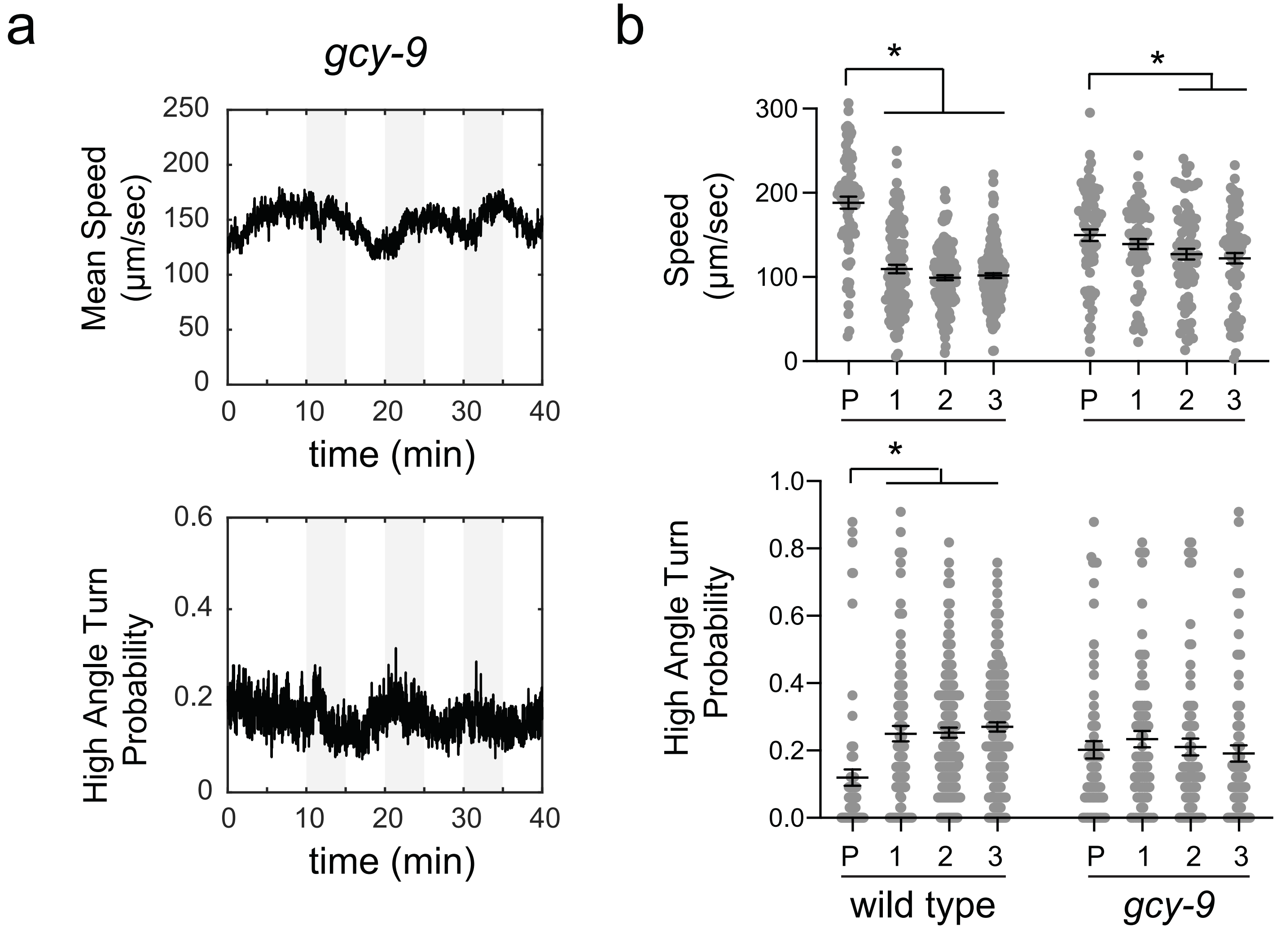

Supplementary Fig. 5 Acute behavioral responses of *gcy-9* mutants to CO_2 stimuli.

a Mean speed and high-angle turn probability changes over time for *gcy-9* mutants. Gray bars indicate CO_2 pulses. **b** Mean speeds and turn probabilities ± SEM of wild type (replotted from Fig. 5) and *gcy-9* mutants during the pre-stimulus period (P) and after each presentation of CO_2 (labeled 1-3). The number of tracks analyzed (*n*) for mean speed during each period (P, 1, 2, and 3) are: 74, 105, 144, and 159 (wild type); 72, 70, 83, and 79 (*gcy-9*). The number of tracks analyzed (*n*) for high angle turn probability during each period (P, 1, 2, and 3) are: 74, 98, 145, and 158 (wild type); 72, 75, 80, and 80 (*gcy-9*). Asterisks indicate $P < 0.05$ for comparisons during each period to P using a Kruskal-Wallis test corrected for multiple comparisons via Dunn's test (*P* values for each comparison of mean speed marked with an asterisk – wild type P vs wild type 1: 4.7×10^{-14}; wild type P vs wild type 2: $< 10^{-15}$; wild type P vs wild type 3: $< 10^{-15}$; *gcy-9* P vs *gcy-9* 2: 0.022; *gcy-9* P vs *gcy-9* 3: 0.010; *P* values for each comparison of high-angle turn probability marked with an asterisk - wild type P vs wild type 1: 5.6×10^{-7}; wild type P vs wild type 2: 3.2×10^{-10}; wild type P vs wild type 3: 6.0×10^{-13}).

Supplementary Figure 6

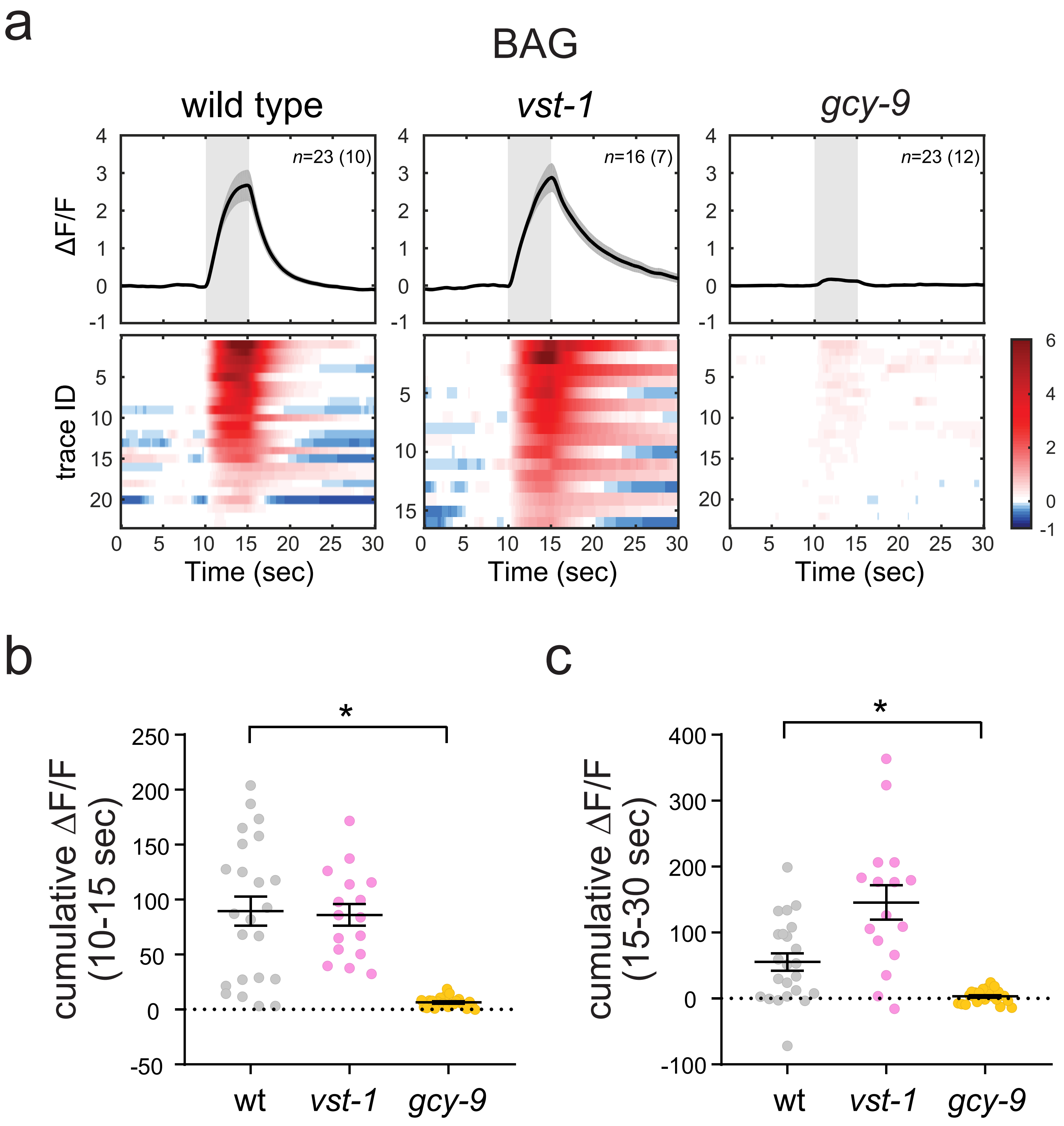

Supplementary Fig. 6 Functional imaging of BAG neuron responses to CO_2 stimuli.

a Fluorescence changes of GCaMP6 expressed in BAG sensory neurons (*Pflp-17::GCaMP6f*) over time in wild-type, *vst-1*, and *gcy-9* animals. The mean signal ± SEM is indicated as a black line and grey shaded region. Individual traces were passed through a 0.5 sec moving-average filter for the plots (raw traces were used for quantification of GCaMP signal in **b** and **c**). **b** Cumulative GCaMP signal (mean ± SEM) between 10 and 15 seconds. Asterisk indicates $P < 0.05$ for comparisons of the mutants to wild type using a Kruskal-Wallis test corrected for multiple comparisons via Dunn's test (*P* value for each comparison – wild type vs *vst*-1: > 0.9999; wild type vs *gcy-9*: $< 3.5 \times 10^{-7}$, n = 23, 16, and 23 for wild type, *vst-1*, and *gcy-9*, respectively). **c** Cumulative GCaMP signal (mean ± SEM) between 15 and 30 seconds. Asterisks indicate $P < 0.05$ for comparisons of the mutants to wild type using a Kruskal-Wallis test corrected for multiple comparisons via Dunn's test (*P* value for each comparison – wild type vs *vst*-1: 0.051; wild type vs *gcy-9*: 0.0062, n = 23, 16, and 23 for wild type, *vst-1*, and *gcy-9*, respectively).

Supplementary Figure 7

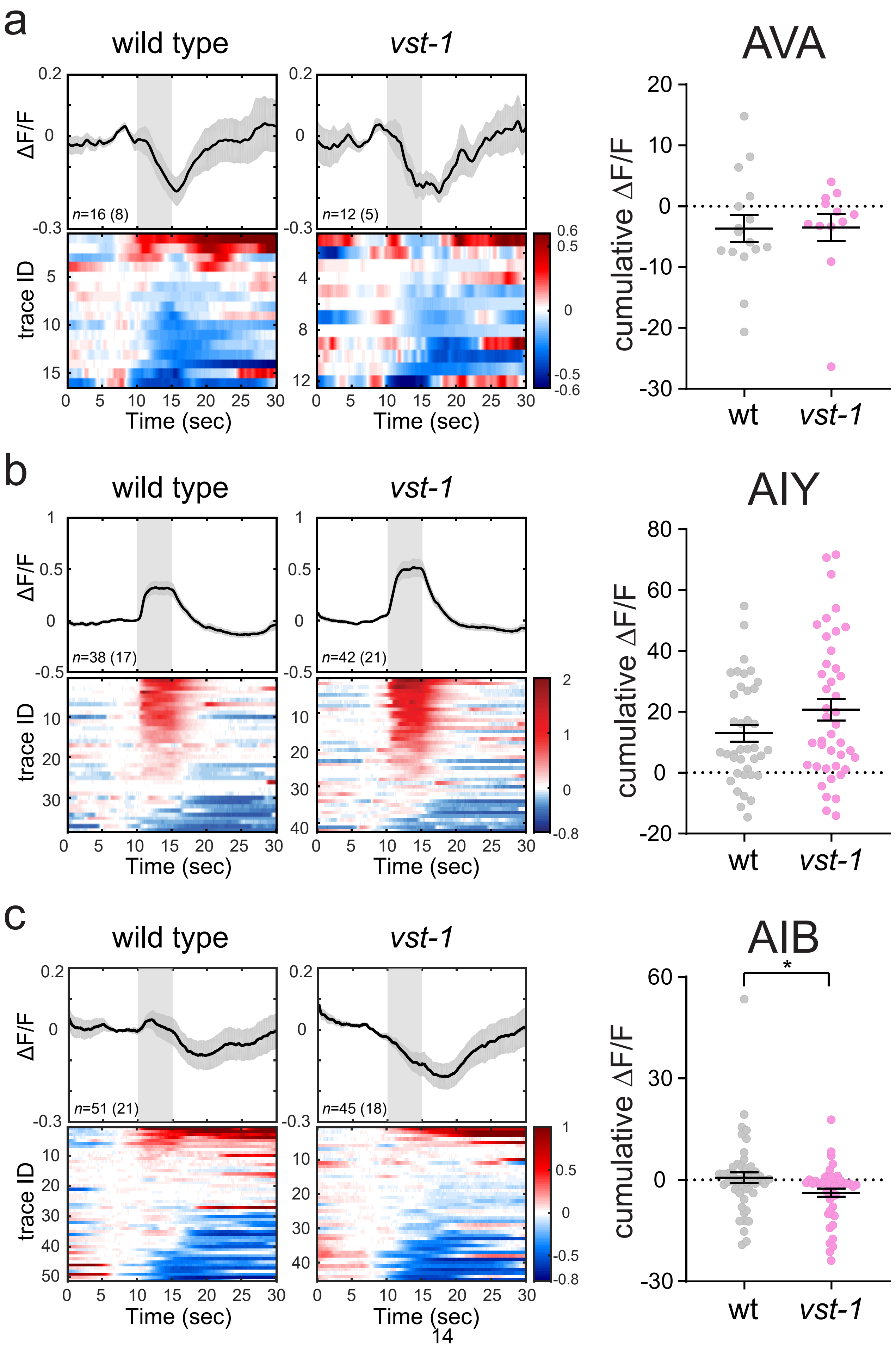

Supplementary Fig. 7 AVA, AIY, and AIB interneuron responses to BAG activation in the wild type and *vst-1* mutants.

a Plots of fluorescence changes of GCaMP6 in the soma of AVA interneurons of *Popt-3::GCaMP6f* animals (mean ± SEM shown as a black line and grey shaded region) (left) and cumulative GCaMP signal (10-15 sec, mean ± SEM) (right). Statistical comparison between wild type and *vst-1* was conducted using a Mann-Whitney test (P = 0.42, two-sided). **b** Plots of fluorescence changes of GCaMP6 in the neurites of AIY interneurons of *Pttx-3::GCaMP6s* animals (mean ± SEM shown as a black line and grey shaded region) (left) and cumulative GCaMP signal (10-15 sec, mean ± SEM) (right). Statistical comparison between wild type and *vst-1* was conducted using a Mann-Whitney test (P = 0.16, two-sided). **c** Plots of fluorescence changes of GCaMP3 in the soma of AIB interneurons of *Pinx-1::GCaMP3* animals (mean ± SEM shown as a black line and grey shaded region) (left) and cumulative GCaMP signal (10-15 sec, mean ± SEM) (right). Statistical comparison between wild type and *vst-1* was conducted using a Mann-Whitney test (P = 0.012, two-sided). For **a-c**, individual traces were passed through a 0.5 sec moving-average filter for the plots (left) but the cumulative signal was quantified (right) using raw traces.

Supplementary Figure 8

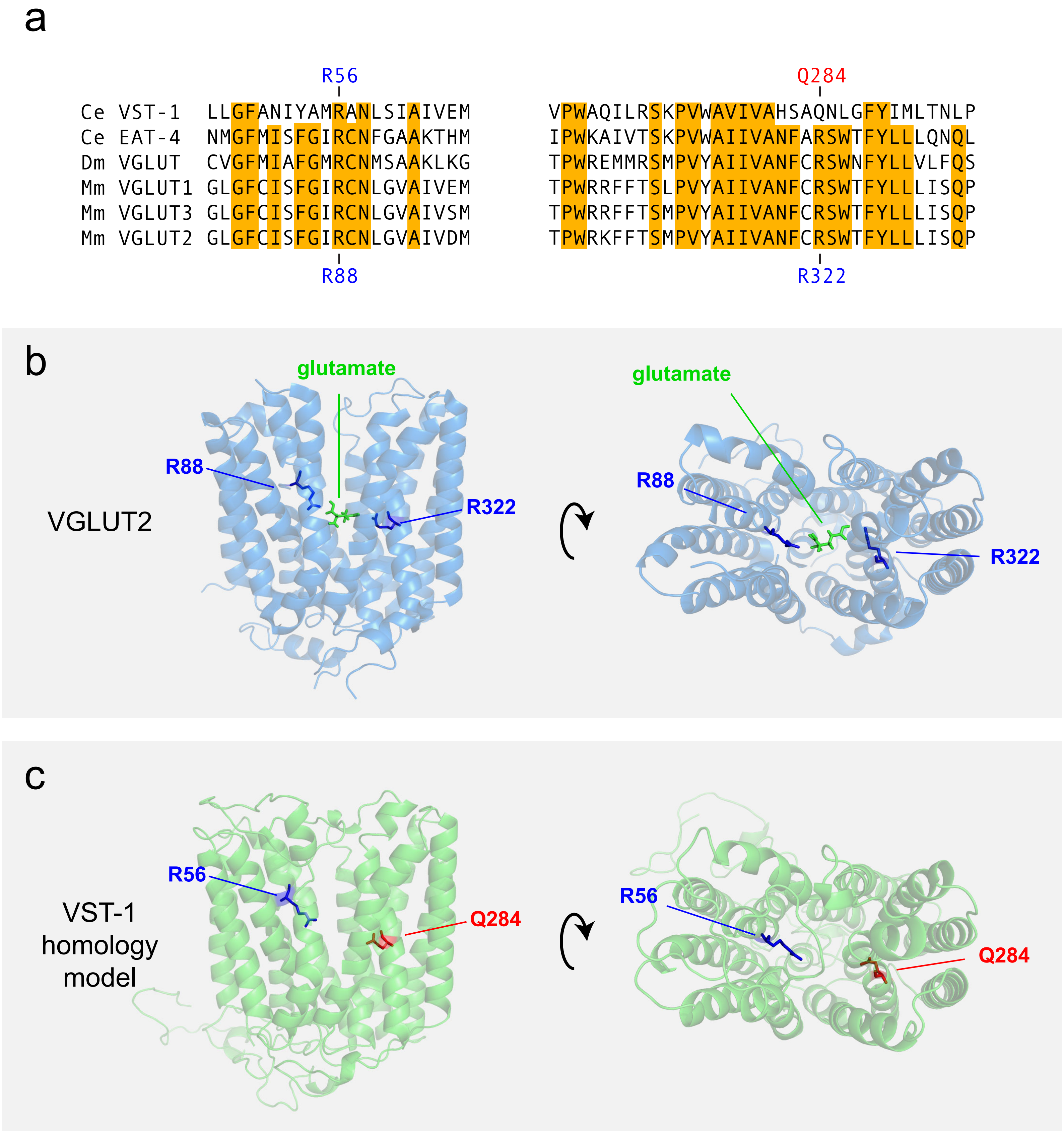

Supplementary Fig. 8 The transporter VST-1 lacks an arginine residue that is critical for glutamate-binding.

a Sequence alignments of regions from the second and seventh transmembrane domains of VST-1 and VGLUTs of *C. elegans* (Ce), *D. melanogaster* (De), and *M. musculus* (Mm). Conserved residues are indicated in orange. Arginine 88 and arginine 322 of VGLUT2, indicated in blue, are critical for VGLUT function and are thought to mediate glutamate-binding[2-4]. The corresponding residues in VST-1 are arginine 56 (blue) and glutamine 284 (red). **b** Structure of VGLUT2 and proposed interactions with glutamate after Li *et al.*[2]. Arginine 88 and arginine 322 are indicated in blue, and glutamate in green. **c** Homology model of VST-1 generated by Phyre2[5] indicating conservation of one arginine required for glutamate transport (R56 in blue) and a substitution of glutamine for the other arginine (Q284 in red).

Supplementary Figure 9

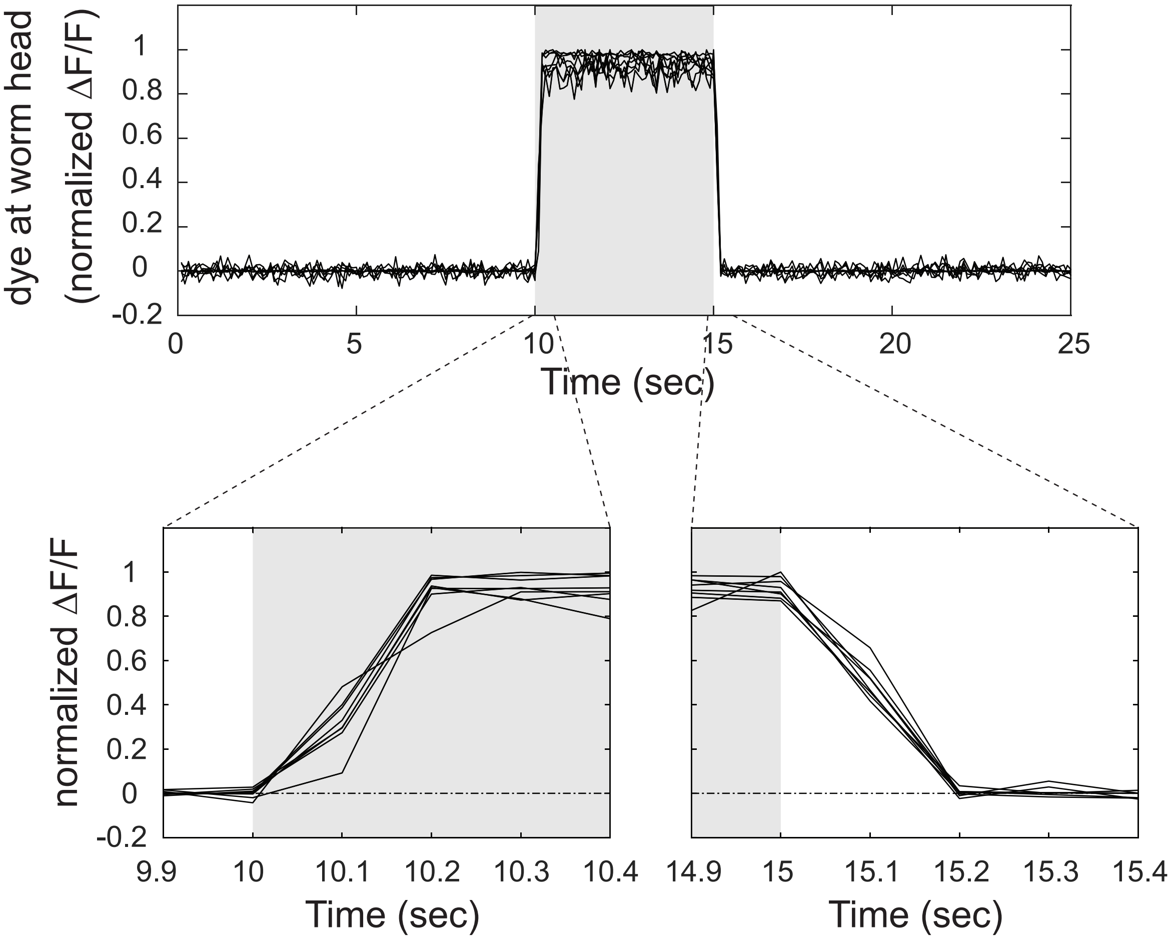

Supplementary Fig. 9 Characterization of solution changes in a microfluidic device driven by positive pressure.

The upper panel shows change of fluorescein signal (ΔF/F) indicating the presence of solution in the channel directed toward the head of the worm (*n* = 8). The lower panels show the plot in the upper panel close to the time of valve switch. Gray indicates the window in which the head of the worm is exposed to fluorescein.

SUPPLEMENTARY TABLES

Supplementary Table 1. Statistical comparisons between different genotypes in Fig. 5.

Statistical comparisons between all genotypes at corresponding periods (P,1,2,3) were conducted using a Kruskal-Wallis test corrected for multiple comparisons by Dunn's test and the *P* values are indicated for each comparison. Bold numbers indicate $P < 0.05$.

Mean Speed

Comparisons	Period			
	**Pre-stimulus**	**Stimulus 1**	**Stimulus 2**	**Stimulus 3**
wild type vs *vst-1*	**<0.0001**	**<0.0001**	**<0.0001**	**<0.0001**
wild type vs ea*t-4*	**<0.0001**	**<0.0001**	**<0.0001**	**<0.0001**
wild type vs ea*t-4; vst-1*	**<0.0001**	**0.0141**	**<0.0001**	**0.0020**
wild type vs glr-*1*	>0.9999	>0.9999	>0.9999	>0.9999
wild type vs *glr-1; vst-1*	>0.9999	0.1834	>0.9999	>0.9999
vst-1 vs *eat-4*	>0.9999	**0.0134**	**<0.0001**	**<0.0001**
vst-1 vs *eat-4; vst-1*	>0.9999	**0.0004**	**<0.0001**	**<0.0001**
vst-1 vs glr-*1*	**<0.0001**	**<0.0001**	**<0.0001**	**<0.0001**
vst-1 vs *glr-1; vst-1*	**<0.0001**	**<0.0001**	**<0.0001**	**<0.0001**
eat-4 vs *eat-4; vst-1*	>0.9999	>0.9999	>0.9999	0.2952
eat-4 vs *glr-1*	**<0.0001**	**0.0138**	**<0.0001**	**<0.0001**
eat-4 vs *glr-1; vst-1*	**<0.0001**	**<0.0001**	**<0.0001**	**<0.0001**
eat-4; vst-1 vs *glr-1*	**<0.0001**	0.4878	**<0.0001**	**0.0204**
eat-4; vst-1 vs *glr-1; vst-1*	**<0.0001**	**<0.0001**	**<0.0001**	**0.0014**
glr-1 vs *glr-1; vst-1*	>0.9999	**0.0164**	>0.9999	>0.9999

Turn Probability

Comparisons	Period			
	**Pre-stimulus**	**Stimulus 1**	**Stimulus 2**	**Stimulus 3**
wild type vs *vst-1*	>0.9999	>0.9999	**0.0013**	**0.0011**
wild type vs ea*t-4*	>0.9999	>0.9999	>0.9999	**0.0167**
wild type vs ea*t-4; vst-1*	>0.9999	>0.9999	**0.0002**	**<0.0001**
wild type vs glr-*1*	0.6504	>0.9999	0.0620	**0.0092**
wild type vs *glr-1; vst-1*	**0.0416**	0.3433	**0.0007**	0.1508
vst-1 vs *eat-4*	>0.9999	>0.9999	**<0.0001**	**<0.0001**
vst-1 vs *eat-4; vst-1*	>0.9999	0.7648	**<0.0001**	**<0.0001**
vst-1 vs glr-*1*	>0.9999	>0.9999	**<0.0001**	**<0.0001**
vst-1 vs *glr-1; vst-1*	0.4635	0.1718	**<0.0001**	**<0.0001**
eat-4 vs *eat-4; vst-1*	>0.9999	**0.0384**	0.0670	**0.0355**
eat-4 vs *glr-1*	**0.0458**	0.9114	>0.9999	>0.9999
eat-4 vs *glr-1; vst-1*	**0.0006**	**0.0063**	0.1482	>0.9999
eat-4; vst-1 vs *glr-1*	>0.9999	>0.9999	>0.9999	0.5437
eat-4; vst-1 vs *glr-1; vst-1*	0.2334	>0.9999	>0.9999	**0.0164**
glr-1 vs *glr-1; vst-1*	>0.9999	>0.9999	>0.9999	>0.9999

Supplementary Table 2. Mutant and transgenic lines used for this study.

Strain	Genotype
FQ306	*gcy-9(tm2816)*
MT6308	*eat-4(ky5)*
VC40514	*vst-1(gk673717)*
VC20397	*vst-1(gk308047)*
FQ1151	*wzEx308[Pflp-17::eat-4 RNAi; Punc-122::mCherry]*
FQ1160	*wzEx315[Pflp-17::vst-1 RNAi; Punc-122::GFP]*
FQ1117	*unc-119(ed3); wzIs134[Pvst-1::vst-1 fosmid::GFP; Punc-122::mCherry; unc-119(+)]; vst-1(gk308047)*
FQ1571	*otIs388[eat-4 fosmid::SL2::YFP::H2B + (pBX) pha-1(+)] pha-1(e2123); wzEx431[vst-1 fosmid::stop::SL2::1xNLS::mCherry::H2B; Punc-122::GFP]*
FQ1137	*vst-1(gk308047); wzEx305[Pvst-1::vst-1::GFP; Pflp-17::mStrawberry]*
FQ2656	*unc-104(e1265); vst-1(gk308047)/+; wzEx305 [Pvst-1::vst-1::GFP; Pflp-17::mStrawberry]*
FQ1167	*wzEx323[Pvst-1::vst-1::GFP; Peat-4::eat-4::mCherry; Punc-122::GFP])*
FQ843	*wzEx204[Pflp-17::iGluSnFR; Punc-122::mCherry]*
FQ891	*eat-4(ky5); wzEx204[Pflp-17::iGluSnFR; Punc-122::mCherry]*
FQ1174	*vst-1(gk673717); wzEx204[Pflp-17::iGluSnFR; Punc-122::mCherry]*
FQ911	*vst-1(gk308047); wzEx204[Pflp-17::iGluSnFR; Punc-122::mCherry]*
FQ1575	*wzEx434[Pgcy-33::snb-1::superecliptic pHluorin; Pflp-17::mStrawberry; Punc-122::mCherry]*
FQ1764	*vst-1(gk308047); wzEx434[Pgcy-33::snb-1::superecliptic pHluorin; Pflp-17::mStrawberry; Punc-122::mCherry]*

FQ1817	*eat-4(ky5); wzEx434[Pgcy-33::snb-1::superecliptic pHluorin; Pflp-17::mStrawberry; Punc-122::mCherry]*
KP4	*glr-1(n2461)*
FQ1277	*glr-1(n2461); vst-1(gk308047)*
FQ1440	*eat-4(ky5); vst-1(gk308047)*
FQ845	*wzEx165[Pflp-17::GCaMP6f; Punc-122::mCherry]*
FQ2110	*vst-1(gk308047); wzEx165[Pflp-17::GCaMP6f; Punc-122::mCherry]*
FQ2143	*gcy-9(tm2816); wzEx165[Pflp-17::GCaMP6f; Punc-122::mCherry]*
CX13440	*kyEx4018[Pinx-1::GCaMP3; Punc-122::dsRed]*
FQ2039	*vst-1(gk308047); kyEx4018[Pinx-1::GCaMP3; Punc-122::dsRed]*
FQ944	*wzEx246[Popt-3::GCaMP6f; Punc-122::mCherry]*
FQ1922	*vst-1(gk308047); wzEx246[Popt-3::GCaMP6f; Punc-122::mCherry]*
FQ2348	*sraEx490[Pttx-3::GCaMP6s]*
FQ2236	*vst-1(gk308047); sraEx490[Pttx-3::GCaMP6s]*
ZC1508	*yxIs19[Pglr-3a::GCaMP3; Punc-122::dsRed]*
FQ2040	*yxIs19[Pglr-3a::GCaMP3; Punc-122::dsRed]; vst-1(gk308047)*
FQ492	*yxIs19[Pglr-3a::GCaMP3; Punc-122::dsRed]; gcy-9(tm2816)*
FQ2178	*yxIs19[Pglr-3a::GCaMP3; Punc-122::dsRed]; gcy-9(tm2816) vst-1(gk308047)*
FQ2176	*glr-1(n2461); yxIs19[Pglr-3a::GCaMP3; Punc-122::dsRed]*
FQ2177	*glr-1(n2461); yxIs19[Pglr-3a::GCaMP3; Punc-122::dsRed]; vst-1(gk308047)*
TV2217	*wyIs93[Pglr-3::mCherry::rab-3; Pglr-3::glr-1::GFP; Punc-122::RFP]; wyEx828[Pglr-3::caspase-3(p12)::nz; Pglr-3::cz::caspase-3(p17); Pglr-3::mCherry; Punc-122::GFP]*

FQ2476	*vst-1(gk308047) wyIs93[Pglr-3::mCherry::rab-3; Pglr-3::glr-1::GFP; Punc-122::RFP]; wyEx828[Pglr-3::caspase-3(p12)::nz; Pglr-3::cz::caspase-3(p17); Pglr-3::mCherry; Punc-122::GFP]*

Supplementary Table 3. Plasmids, fosmids, and primers used for this study.

Name	Description
pJC141	*Pflp-17::vst-1 RNAi sense*
pJC143	*Pflp-17::vst-1 RNAi antisense*
pJC133	*Pflp-17::eat-4 RNAi sense*
pJC135	*Pflp-17::eat-4 RNAi antisense*
pJC75	*Pvst-1::vst-1::GFP* fosmid
pEH62	*vst-1::stop::SL2::1xNLS::mCherry::H2B::stop* fosmid
pJC77	*Pvst-1::vst-1::GFP* fosmid (identical to pJC75)
pJC106	9.6 kb PCR amplicon of *Pvst-1::vst-1::GFP* fosmid (pJC77)
pJC52	*Peat-4::eat-4::mCherry* fosmid
pJC149	14.2 kb PCR amplicon of *Peat-4::eat-4::mCherry* fosmid (pJC52)
pJC7	*Pflp-17::iGluSnFR*
pJC151	*Pgcy-33::snb-1::superecliptic pHluorin*
pKE5	*Popt-3::GCaMP6f*
pJB134	*Pflp-17::mStrawberry*
	Genotyping primers for *vst-1(gk673717)*
JC257	F:ATCGAGACAATAATGGTGCTACGTTTCCCT
JC253	wild type R:TGCCACCACGTGACGTCGCTTAC

JC256	*vst-1* R:CCACCACGTGACGTCGCTCCT
 <br>JC86<br><br>JC81<br><br>JC84	Genotyping primers for *vst-1(gk308047)*<br><br>F:CAATGACGCAAGGAGTTGTGCTCAGTTC<br><br>wild type R:CCATTGGAGGTGCCCATCTGCTAC<br><br>*vst-1* R:CCATTGGAGGTGCCCATCTGGTCT
 <br>JC158<br><br>JC258<br><br>JC159	Genotyping primers for *eat-4(ky5)*<br><br>wild type F:AAGAAGAAGGAAACGAAAACCCGATGC<br><br>*eat-4* F:CCAGCGCGGTGAGTAGTAGATC<br><br>R:TCCATATGGTAGGAGAATATTCAAAAATGCTCCG
 <br>JC277<br><br>JC279<br><br>JC281	Genotyping primers for *glr-1(n2461)*<br><br>wild type F:CGACACCTTTCGGCTCCGACTTG<br><br>*vst-1* F:GCGACACCTTTCGGCTCCGACTTA<br><br>R:TTTTCAAATTTTTCTGAAATCATCTTTATCCACCA
 <br>NR348<br><br>NR349<br><br>NR346<br><br>NR347	Genotyping primers for *gcy-9(tm2816)*<br><br>wild type F:ACTATATAGTTGCGGAAGCACTGG<br><br>wild type R:TGGTTCGAGATCGTACTGCACTCC<br><br>*gcy-9* F:GTGTTAGAAAGGGATCCTGAATCC<br><br>*gcy-9* R:GCAGATATATATGCACTCGGAATGG
 <br>JC178<br><br>JC179	Primers to generate RNAi sense plasmid targeting *vst-1*<br><br>F:atagaacattttcaggaggacccttggctagcagaaaaGTGGTGGCAATTTTGGCGTTGC<br><br>R:cgatgcggagctcagatatcaataccatggtaccAATCCCTCCATGAAGCGAGCAAAAAC
 <br>JC180	Primers to generate RNAi antisense plasmid targeting *vst-1*<br><br>F:aacattttcaggaggacccttggctagcagaaaaAATCCCTCCATGAAGCGAGCAAAAAC

JC181	R:cggccgatgcggagctcagatatcaataccatggtaccGTGGTGGCAATTTTGGCGTTGC
 <br>JC170<br><br>JC171	Primers to generate RNAi sense plasmid targeting *eat-4*<br><br>F:tcaggaggacccttggctagcagaaaaCTTCTAGCTATTCTTGCAAATATGGGATTCATG<br><br>R:tgcggagctcagatatcaataccatggtaccACAAGCCCTTGAGTAATTTGAATGAAAGC
 <br>JC172<br><br>JC173	Primers to generate RNAi antisense plasmid targeting *eat-4*<br><br>F:attttcaggaggacccttggctagcagaaaaACAAGCCCTTGAGTAATTTGAATGAAAGC<br><br>R:gagctcagatatcaataccatggtaccCTTCTAGCTATTCTTGCAAATATGGGATTCATG
 <br>JC206<br><br>JC207	Primers to generate pJC106<br><br>F:AATCAGACTTTATGAGCAACTTGTCAAGATAG<br><br>R:ACTTCACCGTCTTCCTCACCCTTTTC
 <br>JC221<br><br>JC220	Primers to generate pJC149<br><br>F:TGGACTATCAGGAACCGCTGGGTAAG<br><br>R:AGTTGAGCAAGAAGGAAATCAAGGATGTTC

SUPPLEMENTARY REFERENCES

1 Bond, C. S. & Schüttelkopf, A. W. ALINE: a WYSIWYG protein-sequence alignment editor for publication-quality alignments. *Acta crystallographica. Section D, Biological crystallography* **65**, 510-512, doi:10.1107/s0907444909007835 (2009).

2 Li, F. *et al.* Ion transport and regulation in a synaptic vesicle glutamate transporter. *Science* **368**, 893-897, doi:10.1126/science.aba9202 (2020).

3 Chang, R., Eriksen, J. & Edwards, R. H. The dual role of chloride in synaptic vesicle glutamate transport. *eLife* **7**, doi:10.7554/eLife.34896 (2018).

4 Eriksen, J. *et al.* Protons Regulate Vesicular Glutamate Transporters through an Allosteric Mechanism. *Neuron* **90**, 768-780, doi:10.1016/j.neuron.2016.03.026 (2016).

5 Kelley, L. A., Mezulis, S., Yates, C. M., Wass, M. N. & Sternberg, M. J. The Phyre2 web portal for protein modeling, prediction and analysis. *Nature protocols* **10**, 845-858, doi:10.1038/nprot.2015.053 (2015).
