# Supplementary material for: Opponent vesicular transporters regulate the strength of glutamatergic neurotransmission in a C. elegans sensory circuit
Source: Nat Commun. 2021 Nov 3;12:6334. doi: 10.1038/s41467-021-26575-3 (PMC8566550; doi:10.1038/s41467-021-26575-3)
Supplement: Supplementary file 3 — Reporting Summary [file 41467_2021_26575_MOESM3_ESM.pdf]

nature research

Corresponding author(s): Niels Ringstad

Last updated by author(s): Sep 8, 2021

Reporting Summary

Nature Research wishes to improve the reproducibility of the work that we publish. This form provides structure for consistency and transparency in reporting. For further information on Nature Research policies, see our Editorial Policies and the Editorial Policy Checklist.

Statistics

For all statistical analyses, confirm that the following items are present in the figure legend, table legend, main text, or Methods section.

n/a	Confirmed	
☐	☒	The exact sample size (*n*) for each experimental group/condition, given as a discrete number and unit of measurement
☐	☒	A statement on whether measurements were taken from distinct samples or whether the same sample was measured repeatedly
☐	☒	The statistical test(s) used AND whether they are one- or two-sided<br>*Only common tests should be described solely by name; describe more complex techniques in the Methods section.*
☒	☐	A description of all covariates tested
☐	☒	A description of any assumptions or corrections, such as tests of normality and adjustment for multiple comparisons
☐	☒	A full description of the statistical parameters including central tendency (e.g. means) or other basic estimates (e.g. regression coefficient) AND variation (e.g. standard deviation) or associated estimates of uncertainty (e.g. confidence intervals)
☐	☒	For null hypothesis testing, the test statistic (e.g. *F*, *t*, *r*) with confidence intervals, effect sizes, degrees of freedom and *P* value noted<br>*Give P values as exact values whenever suitable.*
☒	☐	For Bayesian analysis, information on the choice of priors and Markov chain Monte Carlo settings
☒	☐	For hierarchical and complex designs, identification of the appropriate level for tests and full reporting of outcomes
☒	☐	Estimates of effect sizes (e.g. Cohen's *d*, Pearson's *r*), indicating how they were calculated

Our web collection on statistics for biologists contains articles on many of the points above.

Software and code

Policy information about availability of computer code

Data collection	Confocal micrographs were taken using ZEN 2012 software (Zeiss, version 8.0) on the Zeiss LSM 700 confocal.<br><br>Electron micrographs were taken using Digital Micrograph software (Gatan, version 1.81.78) on the Philips CM-12 (FEI) transmission electron microscope.<br><br>Movies of iGluSnFR, pHluorin, and GCaMP fluorescent images were acquired using Live Acquisition software (Till Photonics, version 2.5.0.17) on a custom-built inverted fluorescent microscope.<br><br>Videorecordings of worms during acute CO2 exposure were collected on a mounted CCD camera (Unibrain, Fire-I 785b) using a Matlab script (Nihkil Bhatla) according to Ma et al., 2012.
Data analysis	RNASeq data analysis (described in Rossillo et al., 2020)<br>1) Coverage histograms for eat-4 and vst-1 were visualized using the Integrative Genomics Viewer.<br>2) Mean read counts were quantified using Deeptools.<br>3) Fold enrichment and false discovery rate were computed using the DESeq2 package.<br><br>Matlab (versions R2016a and R2020a) was used to analyze and plot cellular glutamate release assay, pH measurements via synaptopHluorin, and GCaMP imaging data.<br><br>A Matlab script (Nihkil Bhatla), described in Ma et al., 2012, was used to identify worms by morphological features and quantify parameters such as instantaneous speed and heading/angle change.

Graphpad Prism (version 8) was used for statistical analysis and plotting of quantified data.

For manuscripts utilizing custom algorithms or software that are central to the research but not yet described in published literature, software must be made available to editors and reviewers. We strongly encourage code deposition in a community repository (e.g. GitHub). See the Nature Research guidelines for submitting code & software for further information.

Data

Policy information about availability of data

All manuscripts must include a data availability statement. This statement should provide the following information, where applicable:

- Accession codes, unique identifiers, or web links for publicly available datasets
- A list of figures that have associated raw data
- A description of any restrictions on data availability

The RNASeq dataset analyzed for this study is available at the accession number GSE137267. All other data used for this study are available from the corresponding author upon request.

Field-specific reporting

Please select the one below that is the best fit for your research. If you are not sure, read the appropriate sections before making your selection.

[x] Life sciences [ ] Behavioural & social sciences [ ] Ecological, evolutionary & environmental sciences

For a reference copy of the document with all sections, see nature.com/documents/nr-reporting-summary-flat.pdf

Life sciences study design

All studies must disclose on these points even when the disclosure is negative.

Sample size

Sample size for each experiment was determined empirically. After initial assessment of variance of a control strain, we determined a sample size that might allow us to compare statistics for different genotypes. We then set sample size for all genotypes to this number.

Data exclusions

We describe criteria for data exclusion and how many measurements were excluded for most experiments in our Methods. To summarize:

Cellular glutamate release assay (iGluSnFR experiments) exclusion criterion:
Cells that moved too much to extract dF/F from ROIs were excluded (movement interferes with accurate measurement).

pH measurements via synaptopHluorin exclusion criteria (criteria 1-5 apply to Figure 3h, criteria 1-2 apply to Extended Data Figure 3d and 3e):
1) pHluorin puncta with very low baseline signal were excluded (these puncta have high fluctuations of dF/F which do not allow accurate measurements of response to KCl).
2) pHluorin puncta that do not exhibit a KCl-evoked increase were excluded (such puncta likely respresent non-synaptic vesicle puncta).
3) pHluorin puncta that do not exhibit a response to NH4Cl or MES were excluded (pH calculation requires a response to both NH4Cl and MES).
4) pH measurements that did not allow calculation of pH were excluded (a small number of measurements resulted in equations that could not be mathematically solved to measure pH).
5) pH measurements that resulted in pH values lower than 5 were excluded (outside of the dynamic range of superecliptic pHluorin).

Videotracking and analysis of acute locomotor response to CO2 exclusion criteria:
1) Objects tracked for less than 30 seconds were excluded.
2) Objects with speeds less than 38.15um/sec were excluded.
These criteria enabled selective exclusion of non-worm objects (extent of exclusion described in Methods).

GCaMP imaging via microfluidic device exclusion criteria:
1) Worms that moved too much to extract dF/F from ROIs were excluded (movement interferes with accurate measurement).
2) Worms that exhibited increases in dF/F during the pre-stimulation period were excluded because such pre-stimulus activity could affect the stimulus-evoked response that was measured.

Replication

Our experimental design required we perform multiple trials over multiple days. Data presented in the manuscript therefore capture within-trial variability and between-trial variability.

Randomization

We randomly selected animals of a single genotype from a plate and conducted experiments according to genotype. Animals were well-fed and grown at 20 degrees C for all experiments. For behavior experiments (CO2 chemotaxis assays and videotracking of acute locomotor response to CO2), we conducted experiments for different genotypes on the same day to minimize the effects of changes in the environment (temperature, humidity, etc.).

Blinding

Investigators did not conduct blind experiments. All experiments were designed to eliminate any requirement for subjective assessment of the data.

Reporting for specific materials, systems and methods

We require information from authors about some types of materials, experimental systems and methods used in many studies. Here, indicate whether each material, system or method listed is relevant to your study. If you are not sure if a list item applies to your research, read the appropriate section before selecting a response.

Materials & experimental systems

n/a	Involved in the study
☐	☒ Antibodies
☒	☐ Eukaryotic cell lines
☒	☐ Palaeontology and archaeology
☐	☒ Animals and other organisms
☒	☐ Human research participants
☒	☐ Clinical data
☒	☐ Dual use research of concern

Methods

n/a	Involved in the study
☒	☐ ChIP-seq
☒	☐ Flow cytometry
☒	☐ MRI-based neuroimaging

Antibodies

Antibodies used

Rabbit anti-GFP, Millipore Sigma, ab3080, 1:10 dilution
Rabbit anti-RFP, ThermoFisher, R10367, 1:20 dilution
15 nm Protein A gold-conjugated secondary antibody, University Medical Center Utrecht Cell Microscopy Core, PA15, 1:50 dilution
5 nm Protein A gold-conjugated secondary antibody, University Medical Center Utrecht Cell Microscopy Core, PA5, 1:50 dilution

Validation

Rabbit anti-GFP was validated by the vendor (https://www.emdmillipore.com/US/en/product/Anti-Green-Fluorescent-Protein-Antibody,MM_NF-AB3080#documentation)
Rabbit anti-RFP was validated by the vendor (https://www.thermofisher.com/order/genome-database/dataSheetPdf?producttype=antibody&productsubtype=antibody_primary&productId=R10367&version=157)
15 nm and 5nm Protein A gold-conjugated secondary antibodies were validated by University Medical Center Utrecht Cell Microscopy Core in Slot et al. 2007, Nature Protocols, 2007

Animals and other organisms

Policy information about studies involving animals; ARRIVE guidelines recommended for reporting animal research

Laboratory animals

Caenorhabditis elegans, hermaphrodites, young adults - strains are listed below:
N2, wild type
FQ306, gcy-9(tm2816)
MT6308, eat-4(ky5)
VC40514, vst-1(gk673717)
VC20397, vst-1(gk308047)
FQ1151, wzEx308[Pflp-17::eat-4 RNAi; Punc-122::mCherry]
FQ1160, wzEx315[Pflp-17::vst-1 RNAi; Punc-122::GFP]
FQ1117, unc-119(ed3); wzIs134[Pvst-1::vst-1 fosmid::GFP; Punc-122::mCherry; unc-119(+)]; vst-1(gk308047)
FQ1571, otIs388[eat-4 fosmid::SL2::YFP::H2B + (pBX) pha-1(+)] pha-1(e2123); wzEx431[vst-1 fosmid::stop::SL2::1xNLS::mCherry::H2B; Punc-122::GFP]
FQ1137, vst-1(gk308047); wzEx305[vst-1::GFP; Pflp-17::mStrawberry]
FQ2656, unc-104(e1265); vst-1(gk308047)/+; wzEx305 [Pvst-1::vst-1::GFP; Pflp-17::mStrawberry]
FQ1167, wzEx323[vst-1::GFP; eat-4::mCherry; Punc-122::GFP])
FQ843, wzEx204[Pflp-17::iGluSnFR; Punc-122::mCherry]
FQ891, eat-4(ky5); wzEx204[Pflp-17::iGluSnFR; Punc-122::mCherry]
FQ1174, vst-1(gk673717); wzEx204[Pflp-17::iGluSnFR; Punc-122::mCherry]
FQ911, vst-1(gk308047); wzEx204[Pflp-17::iGluSnFR; Punc-122::mCherry]
FQ1575, wzEx434[Pgcy-33::snb-1::superecliptic pHluorin; Pflp-17::mStrawberry; Punc-122::mCherry]
FQ1764, vst-1(gk308047); wzEx434[Pgcy-33::snb-1::superecliptic pHluorin; Pflp-17::mStrawberry; Punc-122::mCherry]
FQ1817, eat-4(ky5); wzEx434[Pgcy-33::snb-1::superecliptic pHluorin; Pflp-17::mStrawberry; Punc-122::mCherry]
KP4 glr-1(n2461)
FQ1277, glr-1(n2461); vst-1(gk308047)
FQ1440, eat-4(ky5); vst-1(gk308047)
FQ845, wzEx165[Pflp-17::GCaMP6f; Punc-122::mCherry]
FQ2110, vst-1(gk308047); wzEx165[Pflp-17::GCaMP6f; Punc-122::mCherry]
FQ2143, gcy-9(tm2816); wzEx165[Pflp-17::GCaMP6f; Punc-122::mCherry]
CX13440, kyEx4018[Pinx-1::GCaMP3; Punc-122::dsRed]
FQ2039, vst-1(gk308047); kyEx4018[Pinx-1::GCaMP3; Punc-122::dsRed]
FQ944, wzEx246[Popt-3::GCaMP6f; Punc-122::mCherry]
FQ1922, vst-1(gk308047); wzEx246[Popt-3::GCaMP6f; Punc-122::mCherry]

	FQ2348, sraEx490[Pttx-3::GCaMP6s]<br>FQ2236, vst-1(gk308047); sraEx490[Pttx-3::GCaMP6s]<br>ZC1508, yxIs19[Pglr-3a::GCaMP3; Punc-122::dsRed]<br>FQ2040, yxIs19[Pglr-3a::GCaMP3; Punc-122::dsRed]; vst-1(gk308047)<br>FQ492, yxIs19[Pglr-3a::GCaMP3; Punc-122::dsRed]; gcy-9(tm2816)<br>FQ2178, yxIs19[Pglr-3a::GCaMP3; Punc-122::dsRed]; gcy-9(tm2816) vst-1(gk308047)<br>FQ2176, glr-1(n2461); yxIs19[Pglr-3a::GCaMP3; Punc-122::dsRed]<br>FQ2177, glr-1(n2461); yxIs19[Pglr-3a::GCaMP3; Punc-122::dsRed]; vst-1(gk308047)<br>TV2217, wyIs93[Pglr-3::mCherry::rab-3; Pglr-3::glr-1::GFP; Punc-122::RFP]; wyEx828[Pglr-3::caspase-3(p12)::nz; Pglr-3::cz::caspase-3 (p17); Pglr-3::mCherry; Punc-122::GFP]<br>FQ2476, vst-1(gk308047) wyIs93[Pglr-3::mCherry::rab-3; Pglr-3::glr-1::GFP; Punc-122::RFP]; wyEx828[Pglr-3::caspase-3(p12)::nz; Pglr-3::cz::caspase-3(p17); Pglr-3::mCherry; Punc-122::GFP]
Wild animals	The study did not involve wild animals.
Field-collected samples	The study did not involve samples collected from the field.
Ethics oversight	No ethical approval was required (no human subjects or vertebrate animals were used).

Note that full information on the approval of the study protocol must also be provided in the manuscript.
